# All‐Heat Control of Magnetization Dynamics on Van der Waals Magnets

**DOI:** 10.1002/adma.202501043

**Published:** 2025-06-23

**Authors:** Sumit Haldar, Theodor Griepe, Unai Atxitia, Elton J. G. Santos

**Affiliations:** ^1^ Institute for Condensed Matter Physics and Complex Systems School of Physics and Astronomy The University of Edinburgh Edinburgh EH9 3FD United Kingdom; ^2^ Instituto de Ciencia de Materiales de Madrid CSIC, Cantoblanco Madrid 28049 Spain; ^3^ Higgs Centre for Theoretical Physics University of Edinburgh Edinburgh EH9 3FD UK; ^4^ Donostia International Physics Center (DIPC) Donostia‐San Sebastián Basque Country 20018 Spain

**Keywords:** energy‐efficient process, heat control, ultrafast magnetization dynamics, van der Waals magnets

## Abstract

Heat dissipation in nanomagnetic devices mediated by femtosecond laser excitation constitutes one of the pressing challenges toward energy‐efficient applications yet to be solved. Of particular interest are heterostructures based on 2D van der Waals (vdW) magnets, which benefit from superior interfacial controllability, mechanical flexibility for smart storage platforms, and open‐source for large‐scale production. However, how heat affects the ultrafast magnetization dynamics in such systems, and/or how the spin dynamics can provide alternative pathways for effective heat dissipation have so far been elusive. Here it is shown that the missing link between magnetization dynamics and heat transport is mediated by the thermal conductivity mismatch between the underneath substrate and the vdW magnet. By modeling the laser‐induced ultrafast spin dynamics of three popular vdW materials (CrI_3_, CrGeTe_3_, Fe_3_GeTe_2_) of different electronic characteristics across sixteen substrates of distinct chemical composition, it is found that both the demagnetization and remagnetization timescales are very sensitive to the phonon temperature dynamics through the supporting materials, which defines the heating dissipation efficiency at the interface. The process can be further tuned with the thickness of the vdW magnets, where thin (thick) systems result in faster (slower) magnetization dynamics. It is unveiled that the non‐thermal nature of spin dynamics in vdW heterostructures creates interfacial spin accumulation that generates spin‐polarized currents with dominant frequencies ranging from 0.18 to 1.0 GHz accordingly to the layer thickness and substrate. The findings demonstrate that substrate engineering liaised with the choice of magnetic compounds open venues for efficient spin‐heat control, which ultimately determines the optically excited magnetic characteristics of the vdW layers.

## Graphical abstract

Heat dissipation in nanomagnetic devices mediated by femtosecond laser excitation constitutes one of the pressing challenges toward energy‐efficient information communication technologies. Here it is shown that heat and magnetization play together to determine the ultrafast spin dynamics of van der Waals magnets across any substrates and magnet thickness.

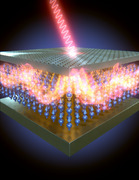

## 1 Introduction

2D van der Waals (vdW) magnets have been attracting significant interest for potential applications in spintronics, due to their low dimensionality, open source, and easy manipulation of their magnetic states via different means toward alternative computing schemes.^[^ ^1^, ^2^, ^3^, ^4^, ^5^, ^6^, ^7^, ^8^, ^9^, ^10^, ^11^, ^12^, ^13^, ^14^ ^]^ Laser technologies offer an exciting pathway toward such control in several forefronts. For instance in terms of ultrafast data processing, where short laser pulses can switch magnetization states within the femtosecond regime, enabling much faster data processing and storage relative to traditional magnetic methods.^[^ ^15^, ^16^, ^17^ ^]^ This translates into energy efficiency, where the energy required to manipulate magnetic states would be a fraction of what is normally required via magnetic field means.^[^ ^16^, ^18^ ^]^ The high precision of lasers at the nanoscale is also crucial for developing high‐density magnetic media where data bits can be promptly accessed without any mechanical contact methods. This strategy allows the reduction of wear and tear of magnetic materials, which can dramatically increase the lifespan of devices.^[^ ^17^, ^19^ ^]^ Moreover, the laser‐spin interactions in vdW magnets provide a realm for fundamental research in truly 2D which has been barely explored up to date. Only recently, there have been reports on the manipulation of spin features in ultrathin vdW magnets and their heterostructures,^[^ ^4^, ^20^, ^21^, ^22^, ^23^, ^24^, ^25^, ^26^ ^]^ which set a practical background for plausible energy‐efficient information storage technologies. An important challenge of any application in laser‐based techniques is how heat is generated and dissipated to nearby environment once ultrashort optical pulses (e.g., 10–40 fs) are applied to the system. While the first part of this issue on the heat generation can be relatively well understood with a variety of approaches based on the flow of energy between separated baths involving electrons, phonons, and spins,^[^ ^4^, ^20^, ^25^, ^27^, ^28^, ^29^, ^30^, ^31^, ^32^, ^33^, ^34^ ^]^ the dissipation scenario becomes a complex phenomenon given the dependence on external substrates and interfacial conditions. Indeed it is unclear the relation between laser‐driven ultrafast magnetization processes in atomically thin vdW layers and the heat flow through underneath substrates.

It is well established that materials with large thermal conductivity tend to dissipate heat more efficiently, thus providing a better media for thermal management for non‐magnetic compounds. Notably, control of ultrafast heat dissipation has proven to be key in reaching the fundamental temporal^[^ ^35^ ^]^ and spatial^[^ ^36^ ^]^ limits of magnetic all‐optical switching.^[^ ^15^ ^]^ The coupling between the spin system with its external environment allows exchange of angular momentum which can either cool down the spins, which promotes remagnetization, or warm it up inducing demagnetization. The exact heating process would depend on the polarization of the transferred angular momentum, and how the magnet responds to the laser‐pulse at a given fluence. If the coupling with the external substrate is sizeable, for instance, through chemical bonds at the interface, angular momentum dissipation may occur, leading to fast demagnetization, as observed in thin‐films deposited on various substrates.^[^ ^37^, ^38^ ^]^ Achieving similarly fast remagnetization is typically challenging, as the system's cooling rate with the environment requires much longer timescales, which limits applications requiring rapid spin dynamics in both directions (demagnetization and remagnetization). Whether the substrate can control or tune the intrinsic spin dynamics within the medium or provide additional properties to the system remains yet to be reported.

Here, we investigate the magnetization dynamics of semiconducting Cr_2_Ge_2_Te_6_ (CGT), insulating CrI_3_, and metallic Fe_3_GeTe_2_ (FGT) deposited on top of various substrates widely used in lab setups: SiO_2_, hBN, Bi_2_Te_3_, ZnO, MoSe_2_, MoS_2_, WS_2_, WSe_2_, ITO, phosphorene, silicene, graphene, black phosphorus, AIN, stanene, Al_2_O_3_. We reveal that the timescales of demagnetization and remagnetization can be engineered on‐demand by the right choice of substrates. We demonstrate that phonon temperature dynamics are crucial in determining how heat dissipates from the 2D magnets to the adjacent system, which acts as an efficient energy reservoir while also enabling manipulation of ultrafast spin dynamics in the vdW layers. We found that the magnetization recovery scales roughly with the inverse of the sample thickness, allowing quicker (slower) magnetization recovery in heterostructures with thinner (thicker) magnetic layers. These magnetization timescales show a universal behavior in relation to the substrate thermal conductivity indicating control on the type of magnetization process (either type‐I or type‐II) after the laser excitations.^[^ ^32^ ^]^ Furthermore, we unveil the non‐thermal nature of the spin dynamics where a transient spin accumulation is generated at the interface, inducing spin‐polarized currents in the GHz regime. Our findings suggest several ingredients on the controllability of vdW magnets toward energy‐efficient ultrafast spintronics not present in regular magnetic thin‐films and bulky materials.

## 2 Results

**Figure 1:**
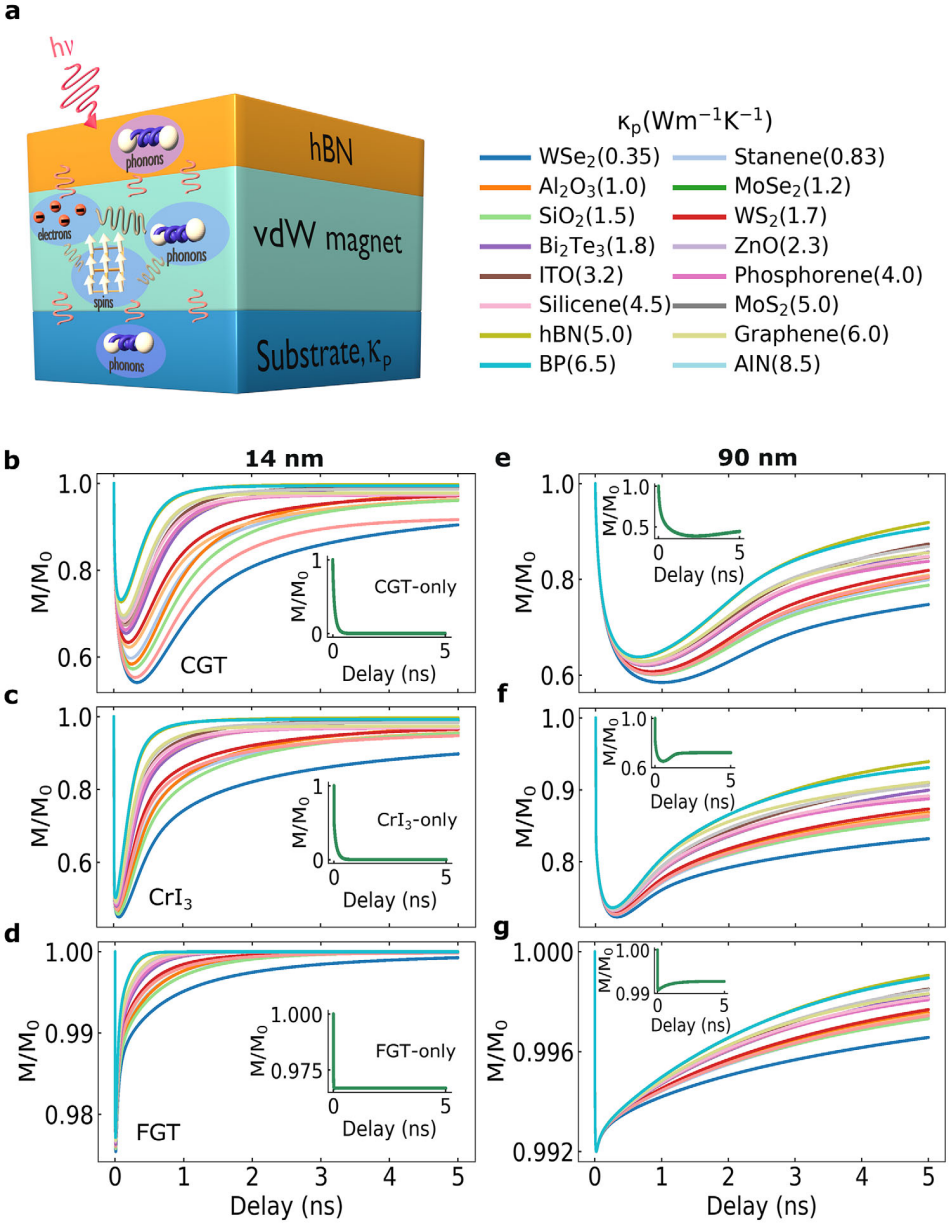
Substrate‐driven dynamics. a) Schematic of the laser‐driven hBN/vdW magnet/substrate heterostructure. The thicknesses are 14 nm for hBN, and 300 nm for the substrates. Different substrates were considered with their respective thermal conductivity coefficient κ_p_ (W m^−1^ K^−1^). The reservoirs (spin, phonons, electrons) are illustrated to indicate how they play together in the heat generation and dissipation into the cap layer and substrate. All systems were excited with a laser fluence of 0.3 mJ cm^−2^ and a wavelength of 800 nm (1.55 eV) at 6 K. b–d) M/M_0_ versus delay time for CrGeTe_3_ (CGT), CrI_3_ and Fe_3_GeTe_2_ (FGT), respectively, at 14 nm thickness. Insets show M/M_0_ for the vdW magnets only without any underneath substrates. e–g) Similar as (b‐d) but with a thickness of 90 nm for the vdW magnets.

To understand the influence of thermal diffusion on the time responses of vdW heterostructures, we investigated the normalized magnetization dynamics (M/M_0_) of the aforementioned magnets encapsulated with insulating hBN layers (14 nm) and deposited on top of different substrates of similar thickness (300 nm) (**Figure** **1**a). We selected the substrates in terms of a wide variation of their thermal conductivity coefficients κ_p_ ranging from 0.35 W m^−1^ K^−1^ (WSe_2_) up to 8.5 W m^−1^ K^−1^ (AIN) to cover most of the surfaces used in vdW junctions. We used two thicknesses for the magnets (14 nm, 90 nm) to highlight the differences in the magneto‐thermal response arising from the sample volume relative to the substrate. Magnetization dynamics are calculated using a time‐extended, layer‐resolved microscopic three temperature model(M3TM)^[^ ^30^ ^]^ taking into account the full vdW junction (see Experimental Section for details). The penetration depth of the pulse was calculated at 30 nm using Abeles' matrix method^[^ ^39^ ^]^ with refractive indices taken from literature.^[^ ^40^, ^41^, ^42^ ^]^

Figure 1b–g show that as soon as the laser is applied into the system a rapid demagnetization and remagnetization take place at the magnet with a strong dependence on its thickness, electronic characteristics (semiconductor, metal), and underneath substrate included. For thin systems (14 nm), CGT displays the broadest variations of the demagnetization and remagnetization processes among the considered magnets relative to the choice of substrate (Figure 1b–d). A gradual modification of the magnetization dynamics is observed as we move from CGT to FGT, with CrI_3_ being at an intermediate point where the different substrates seem to contribute almost similarly to demagnetisation. FGT presents the quickest changes on M/M_0_ for the applied fluence with a maximum demagnetization just a fraction of the others systems. It is important to remark that the Curie temperature (*T*_*C*_) of FGT (*T*_*C*_ = 220 K) is much higher than those at CGT (*T*_*C*_ = 65 K) and CrI_3_ (*T*_*C*_ = 61 K), thus reflecting in higher fluences to reach larger demagnetization (see Figure S1, Supporting Information for additional results).

One of the key observations in the spin dynamics displayed in Figure 1b–d is how the magnitude of κ_p_ for the substrates determines the overall behavior. As κ_p_ decreases, the demagnetization amplitude increases, and the dip at M/M_0_ corresponding to maximum demagnetization shifts to longer time delays, and vice‐versa. For instance, for heterostructures where the underneath substrate has a small κ_p_ (e.g., hBN/CGT/WSe_2_), the heat generated by the laser excitation into the magnet takes longer to be dissipated thus inducing higher demagnetization of the layer (M/M_0_ ≈ 57%) followed by longer time delays for magnetization recovery. Opposite process occurs for large values of κ_p_ (e.g., hBN/CGT/AIN) which fast demagnetization and remagnetization appears. However, the amount of demagnetization (M/M_0_ ≈ 74%) is not the same since a fast heat transfer to the substrate takes place. We also noticed that the magnitudes of κ_p_ are generally low for CGT, CrI_3_, and FGT (see Table S1, Supporting Information) suggesting non‐efficient heat transfer at the vdW magnet per se but an intrinsic dependence on external substrates. Moreover, the variation of M/M_0_ with time is easily tunable with the thickness of the vdW layer, which acts as an additional knob on the control of their spin dynamics. For 90 nm thick magnets (Figure 1e–g), larger time delays can be noticed across the systems with modification of both demagnetization and remagnetization. Interestingly, if no substrates are considered (insets in Figure 1b–g) the demagnetization occurs at a much faster rate inducing M/M_0_ = 0 at very short delay times, and take substantially longer to recover (>>5 ns). Variation of the laser fluence used in the systems do not change the overall picture (Figures S1 and S2, Supporting Information). This indicates the critical role of the underneath substrate on the control of the magnetization processes on vdW magnets.

These results raised an outstanding question such as how quantitatively the relevant timescales of demagnetization and remagnetization depend on the mismatch between the thermal properties of the vdW magnet and the substrate. To determine so, we fitted the normalized magnetization data in Figure 1 using the analytical solution of the M3TM in the low fluence regime:^[^ ^43^, ^44^ ^]^

(1)

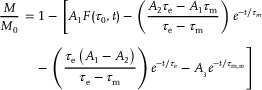

where τ_e_ is a time constant for the rapid demagnetization caused by electron–phonon coupling (*g*_*e* − *p*_), τ_*m*_ represents the slower demagnetization due to spin relaxation after electron–phonon equilibration, τ_m, re_ describes the remagnetization timescale. The constants *A*_1_, *A*_2_, and *A*_3_ represent the demagnetization amplitude after electron–phonon‐spin equilibration, the demagnetization amplitude by the initial increase in electron temperature and the state filling amplitude, respectively. The function *F*(τ_0_, *t*) describes the heat diffusion to the substrate with τ_0_ being the corresponding time‐scale. Tables S3‐S6 and Figures S16–S27 (Supporting Information) provide the full description of the fitted data for all substrates and magnets.

**Figure 2:**
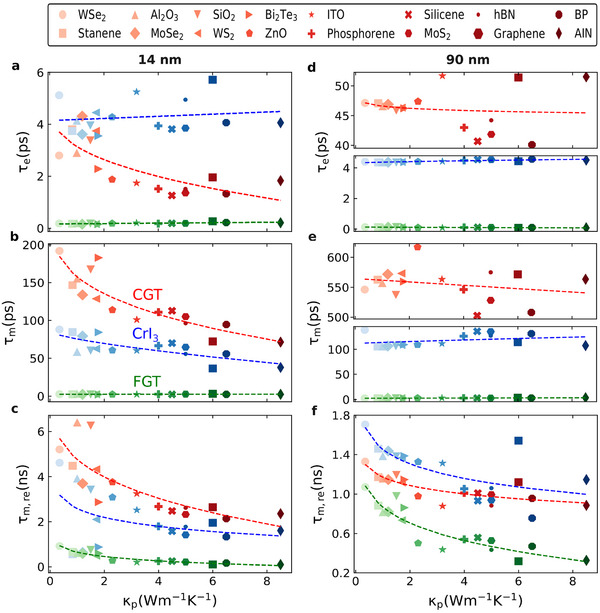
Thermal conductivity as a descriptor of the magnetization dynamics a–c) Time coefficients versus thermal conductivity κ_p_ of the substrates representing the rapid demagnetization (τ_e_), slower demagnetization (τ_m_), and remagnetization (τ_m, re_), respectively, extracted from the magnetization dynamics in Figure 1b–d. The thickness is 14 nm for CGT, CrI_3_ and FGT. d–f) Similar as (a–c), but for magnetization dynamics of Figure 1e–g with a thickness of 90 nm for the vdW magnet. Dashed lines are fitting curves to the calculated symbols for the different substrates.

**Figure** **2** shows a strong dependence of the different timescales τ_e_, τ_m_, and τ_m, re_ with κ_p_, which is sensible to the electronic character of the vdW magnet and its thickness. For thin systems, the correlation is more pronounced for τ_*m*_ and τ_m, re_ (Figure 2b,c) as different power‐law curves in the form of $\alpha \kappa_{p}^{n}+\tau^{0}$ (where α, *n* and τ^0^ are fitting coefficients) can fit the data. Exponents *n* in the range of 0.275 − 0.361 are found for all three magnets (see Table S7, Supporting Information for additional details). The rapid demagnetization τ_e_ tends to follow similar trend with κ_p_ for CGT but it is mainly flat for CrI_3_ and FGT (Figure 2a). This difference in the behavior of τ_e_ for CGT among the three magnets is due to a combination of different ingredients (e.g., Debye temperature, thermal conductivities, etc.) but in particular on the value of *g*_*e* − *ph*_ that rapidly equilibrates the electrons with the phonons. In the case of FGT, which has the largest value of *g*_*e* − *ph*_ among the considered 2D magnets, τ_e_ barely changed across all substrates with an off‐set coefficient τ^0^ = 0.16 ps describing well the overall behavior, indicating that electrons and phonons equilibrate on timescales so quick that heat flow to the substrate has little to no effect on this process. It is noteworthy that the laser fluence also contributes to the difference of τ_e_ among the three magnets since the demagnetisation is not the same, thus affecting the data fitting. A different magnetic response is noticed as the thickness of the magnets is enlarged (Figure 2d–e). Both τ_e_ and τ_m_ show weak dependence on the type of substrate used and display slightly constant magnitudes. This distinct behavior is observed due to the low thermal transport through thick magnets, leading to a non‐effective coupling with the support. Only at the nanosecond domain when the heat starts being dissipated through the substrate a clear dependence is present which appears at τ_m, re_ due to the long recovery times (Figure 2f). These results demonstrated what timescales are more relevant relative to a particular thickness used.

**Figure 3:**
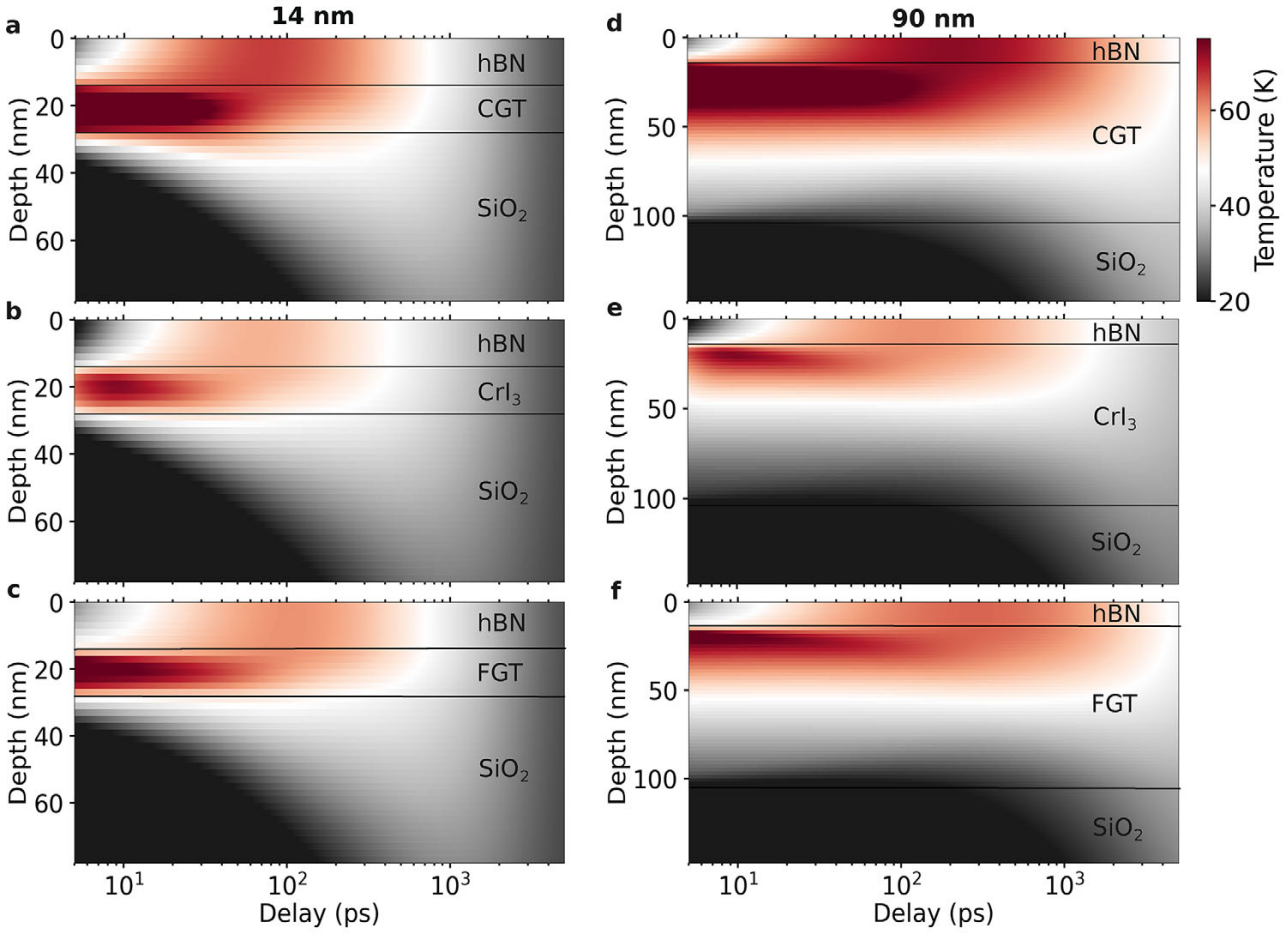
Laser‐induced changes in phonon temperature dynamics. a–c) Phonon temperature dynamics across the vdW heterostructures composed by hBN/CGT/SiO_2_, hBN/CrI_3_/SiO_2_, and hBN/FGT/SiO_2_, respectively. A similar thickness of 14 nm is used for the vdW magnets, with 14 nm for hBN and 300 nm for SiO_2_. d–f) Similar as (a–c) but at 90 nm thickness for the vdW magnets. The laser fluence is 0.3 mJ cm^−2^. Additional substrates are analyzed in Figures S4–S15 (Supporting Information).

In order to gain deeper insight into the demagnetization and remagnetization processes, we investigated the phonon temperature dynamics induced by the optical excitation at the vdW heterostructures considering the penetration depth of the heat across the interfaces (**Figure** **3**). We initially concentrated on SiO_2_ as the substrate composing the junction due to its wide popularity on vdW devices, and to provide a general picture of the contribution of phonons on the heat transfer. Figures S4–S15 (Supporting Information) provide a full analysis on the other substrates investigated. Figure 3 shows that despite of the initial conclusions that underneath substrates are pivotal on the heat transfer from the vdW magnets, hBN also shows a significant heat dissipation in the heterostructures. The laser pulse induced a fast variation of both electron and phonon temperatures, which started equilibrating within the first few picoseconds after the excitation (Figure S3, Supporting Information). Since most of the electronic contributions for the specific heat of the substrates and cap layer are negligible, most of the heat transfer is mediated by the phonon bath. After this initial period, the regime is dominated by the interfaces defined by magnet/hBN and magnet/substrate. Because hBN has significantly higher thermal conductivity and heat capacity than the vdW magnets and SiO_2_ (Table S2, Supporting Information), an asymmetric variation of the phonon temperature is observed, e.g., more heat is transferred to hBN than on SiO_2_. This tends to be modified as time evolves.

For thin magnets (Figure 3a–c), high temperatures are mainly present on hBN within ≈1 ns, followed by a fast cooling towards heat transfer to SiO_2_. Some competition between the amount of heat conducted into hBN and the substrate might be noticed if the magnitude of κ_p_ for both systems is similar. Nevertheless, the presence of the underneath substrate still determines the overall behavior either accelerating or slowing down the magnetization dynamics. As the thickness of the magnets is incremented (Figure 3d–f), most of the phonon temperature becomes localised at the hBN/magnet interface with a large spread throughout the timescale analysed. A slow relaxation of the heat across the vdW magnet is noticed, which only reached the substrate at delay times beyond 1 ns, owing to the low out‐of‐plane component of the highly anisotropic thermal conductivity of the magnetic compounds. This induces a slow remagnetization (Figure 1e–g) of the magnet due to the high thermal disorder. Similar phenomena is observed for the other substrates, which indicated the universal character of this effect.

Having established that the timescales of magnetization dynamics can be controlled by the choice of magnetic material and substrate, we now turn toward the significance of the magnetization dynamics on the thermalization time of the spin system and how additional effects can be generated at the vdW interfaces. In general, a thermalized spin system **S**_*i*_ at an electron temperature *T*_*e*_ follows a distribution given by $f\left(S_{i}\right)∼\exp\left(-H\left(S_{i}\right)/k_{B}T_{e}\right)$, where *k*_B_ is the Boltzmann constant. In the mean‐field approach, the equilibrium magnetization *m*_eq_(*t*) of a thermalized spin system is found through the self‐consistent solution of:

(2) $$\begin{array}{c} m_{\mathrm{eq}}=B_{S}\left(m_{\mathrm{eq}},T_{e}\right) \end{array}$$

where $B_{S}=\frac{2S+1}{2S}\coth\left(3\frac{2S+1}{2S}\frac{T_{C}}{T_{e}}m\right)-\frac{1}{2S}\coth\left(\frac{3}{2S}\frac{T_{C}}{T_{e}}m\right)$ is the Brillouin function for effective spin *S*, evaluated at a given T_*e*_ and *m*. By evaluating *m* linearly in deviations *m* = *B*_*S*_(*m*, *T*_*e*_) + δ*m* one finds:

(3) $$\begin{array}{c} \frac{dm}{dt}∼-R\;\left(1+B_{S}^{\prime }\right)\;\delta m \end{array}$$

where *R* is the magnetization rate parameter and $B_{S}^{\prime }$ is the derivative of the Brillouin function with respect to *m*. Specifically in CGT and CrI_3_, where *R* is very small (≈0.01 1/*ps*), the magnetization dynamics are driven by large deviations of δ*m* and thus a non‐thermal equilibrium state. Indeed, the non‐thermal magnetization dynamics drive the formation of transient spin accumulations Δµ_*s*_∝*dm*/*dt*∝*j*_*s*_, and thus the generation of current densities (*j*_*s*_) in regular thin‐films.^[^ ^45^, ^46^ ^]^

**Figure 4:**
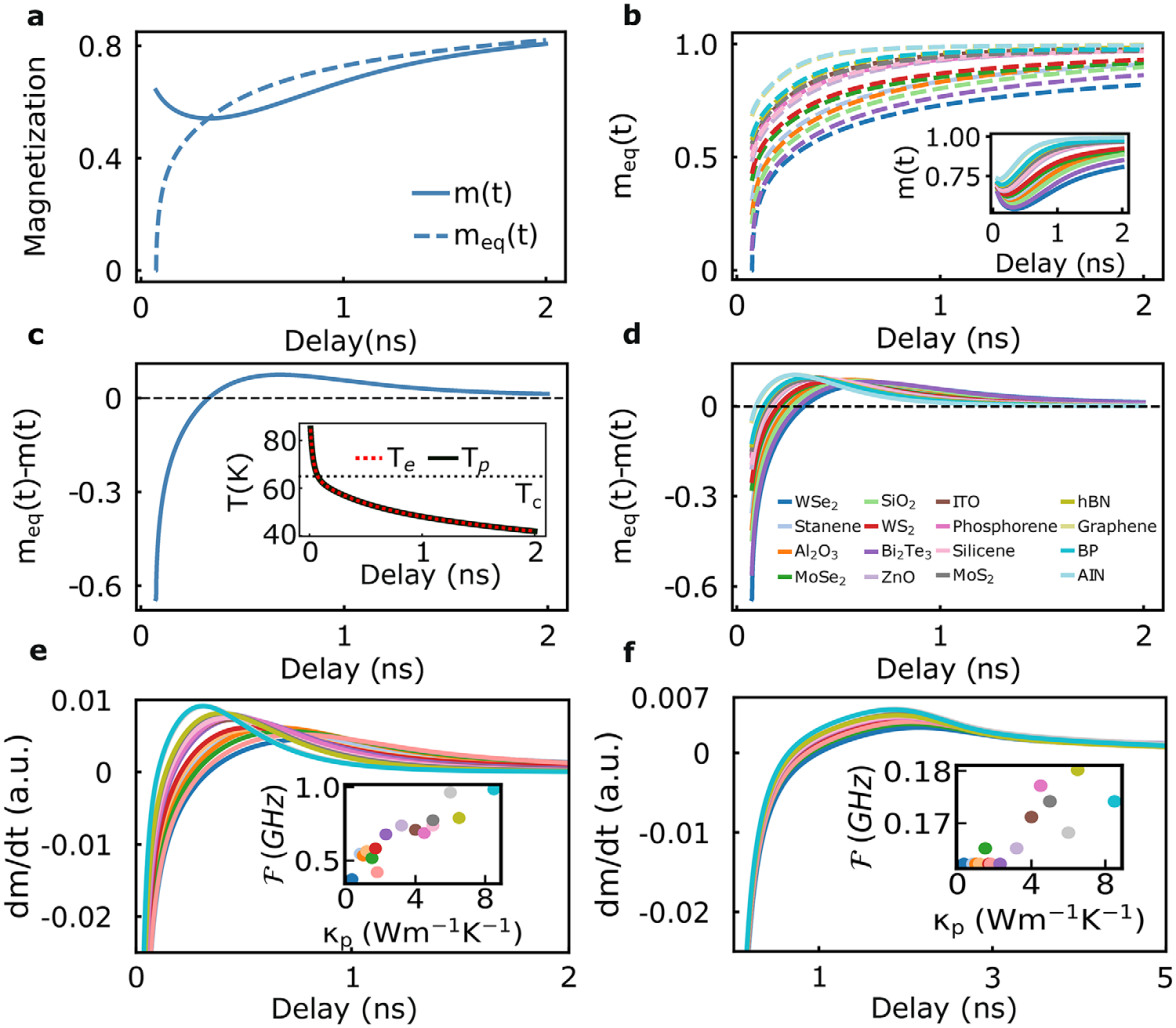
Non‐equilibrium thermal behavior. a) Temporal variation of the average magnetization dynamics *m*(*t*) and equilibrium magnetization *m*_eq_(*t*) computed through M3TM and Brillouin function (Equation 2), respectively, for hBN/CGT/WSe_2_. b) Similar as in (a) but including all substrates. The inset shows *m*(*t*) which is separated from *m*_eq_(*t*) for clarity. c) Difference *m*_eq_(*t*) − *m*(*t*) extracted from (a) as function of time. The inset shows the corresponding evolution of its electron (T_*e*_) and phonon (T_*p*_) temperatures. The Curie temperature (T_*c*_) for CGT is highlighted with the dashed line. d) Similar as in (c) but for all substrates. Labels apply for all panels. e) $\frac{dm}{dt}$ versus time for all junctions of the form hBN/CGT/X, where X corresponds to all substrates. The inset shows the dominant frequencies F(ω) extracted for each substrate where CGT makes an interface. A 14 nm thick CGT is used for (a‐e). f) Similar as in (e) but for a 90 nm thick CGT system.

Intriguingly, we observed that there is a general behavior of the spin accumulation or spin voltage in magnetic vdW heterostructures with the consequent generation of spin‐polarized currents. **Figure** **4**a illustrates the non‐thermal nature of spin dynamics in hBN/CGT/WSe_2_ junctions using CGT samples with a thickness of 14 nm (see Figure S28, Supporting Information for 90 nm thick CGT). The average magnetization *m*(*t*) calculated from the M3TM differs significantly from the equilibrium magnetization *m*_eq_(*t*) in a time range of up to ≈1.5 ns (Figure 4a). A similar trend is also noticed across all substrates considered (Figure 4b) with small variations due to specific characteristics of the materials. This phenomenon can be explained in terms of the temperature variations involved. When *T*_*e*_ exceeds the Curie temperature *T*_*C*_ just after the laser excitation (inset in Figure 4c) *m*_eq_(*t*) drops to zero and begins to recover as *T*_*e*_ falls below *T*_*C*_. This recovery is reflected in the magnetization *m*(*t*), which lags behind the equilibrium magnetization *m*_eq_(*t*), highlighting the non‐thermal state of the spin dynamics. This lag occurs because M3TM assumes a finite rate of spin flips during electron–phonon scattering events, which govern the magnetization dynamics.

The distinguishing variations of *m*(*t*) and *m*_eq_(*t*) are much notorious as their difference (*m*_eq_(*t*) − *m*(*t*)) is taking into account relative to the thermalized regime (dashed line in Figure 4c). The greatest value of *m*_eq_(*t*) − *m*(*t*) is when T_*e*_ and T_*p*_ surpasses *T*_*C*_ at the sub‐nanosecond regime, which diminishes as the spin system gradually equilibrates with the electron and phonon reservoirs. As the temperature reduces further below *T*_*C*_ *m*_eq_(*t*) − *m*(*t*) tends to zero at longer times ≈2 ns with a similar evolution to all substrates investigated (Figure 4d). One of the direct implications of these results is that *dm*/*dt* generates interfacial spin‐polarized currents (Figure 4e–f) at a timescale long enough to be measured through optical pump‐probe techniques.^[^ ^45^, ^47^ ^]^ As *dm*/*dt*∝*j*_*s*_ (see Section S8, Supporting Information for details), its Fourier transform results in the frequency spectrum of the electromagnetic pulse created, thus the maximum where the current is emitted. Depending on the 2D magnet and its thickness, and the substrate selected, the dominant frequencies can range from 1.0 − 0.18 GHz (insets in Figure 4e–f). It is worth mentioning that in 3d transition metals deposited on Pt substrates^[^ ^45^ ^]^ Type‐I spin‐dynamics is established, thus remagnetization occurred after a few picoseconds. However, in the vdW systems studied here, we noticed Type‐II dynamics and diffusion driven remagnetization after hundreds of ps reaching ns‐regime, which is about three orders of magnitude slower. Because the spin currents and thus electromagnetic wave pulses scale with *dm*/*dt*, the time at which demagnetization and remagnetization phases meet determines the dominant frequency of the electromagnetic pulse that can be excited.

## 3 Discussion

The discovery of the crucial role of thermal conductivity on demagnetization and remagnetization processes in ultrathin vdW magnets creates exciting avenues for efficient heat dissipation in real devices platforms. It provides a single knob on the control of the complex interplay between ultrafast laser pulses, underneath substrates, electronic properties of the vdW magnet and its thickness with high accuracy. Our results revealed how phonon temperature impacts the magnetization dynamics in three popular vdW magnets (CGT, CrI_3_, and FGT) contingent on substrate selection. The extracted timescales exhibit a universal behavior relative to the substrate's thermal conductivity. This enhanced understanding provides several promising routes for the optimization of material properties toward tailored performance for specific implementations. Our findings emphasize the necessity of efficient thermal management strategies to maintain device functionality where rapid and reversible control of magnetic states is essential in information storage technologies. The substrate selection in this case becomes as important as the vdW magnet to be utilized, which provides pathways for energy‐efficient protocols as described in our work. Such an approach is normally unreachable in more traditional magnetic thin‐film systems, where lattice mismatch and device fabrication impose serious constrains in the substrate selection that is not present in vdW technology. The real challenge however is to engineer the right combination of vdW magnet and substrate where fast heat dissipation can be achieved with specific magnetization dynamics and current generation but at low energy usage. The guidelines included here provided a step ahead in spin‐heat interfacial engineering.

## 4 Experimental Section

The theoretical approach to understanding ultrafast magnetization dynamics was based on the microscopic three temperature model (M3TM).^[^ ^4^, ^8^, ^20^, ^25^ ^]^ In this framework, the ultrafast laser pulse *S*(*z*, *t*) interacted with the electronic subsystem in the electric dipole approximation, where electrons near the Fermi energy get excited above the bandgap. Upon fast thermalization of the electron system by Coulomb scattering, the laser pulse causes a substantial elevation in the electronic temperature *T*_*e*_. Electron–phonon scattering processed allow an exchange of energy and equilibration of *T*_*e*_ and the phonon bath, characterized by it's temperature *T*_*p*_. Depth dependent laser absorption leads to temperature gradients in the sample that cause diffusion processes in both baths. Energetic cost of demagnetization gets compensated by the electronic system. All interactions in this thermal picture can be captured through:

(4) $$\begin{array}{ccc} C_{e}\frac{dT_{e}}{dt} & = & \frac{\partial }{\partial z}\left(k_{e}\left(\frac{T_{e}}{T_{p}}\right)\frac{\partial T_{e}}{\partial z}\right)+g_{e-p}\left(T_{p}-T_{e}\right)+S\left(z,t\right)+{\dot{Q}}_{\mathrm{es}} \end{array}$$

(5) $$\begin{array}{ccc} C_{p}\frac{dT_{p}}{dt} & = & \frac{\partial }{\partial z}\left(k_{p}\frac{\partial T_{p}}{\partial z}\right)-g_{e-p}\left(T_{p}-T_{e}\right) \end{array}$$

where ${\dot{Q}}_{e-s}$ accounts for the finite energy cost (gain) of a spin‐flip and couples it to the electron dynamics, defined as:

(6) $$\begin{array}{c} {\dot{Q}}_{e-s}=Jm\dot{m}/V_{\mathrm{at}} \end{array}$$

where *V*_at_ denotes the mean atomic volume, and the exchange energy *J* is calculated through the mean field approximation by $J=3\frac{S^{2}}{S(S+1)}k_{B}T_{C}$.

The laser pulse power was characterized in time and space by a Gaussian function centered around *t*_0_ and duration σ and Lambert‐Beer absorption along the z‐axis, respectively:

(7) $$\begin{array}{c} S\left(z,t\right)=\frac{S_{0}}{\lambda }\;e^{-\frac{{\left(t-t_{0}\right)}^{2}}{2\sigma^{2}}}\;e^{-z/\lambda } \end{array}$$

The electron heat capacity in the free electron picture was described by the Sommerfeld approximation *C*_e_ = γ_e_ *T*_e_ and the lattice specific heat was computed with the Einstein model, where:

(8) $$\begin{array}{c} C_{p}=C_{p\infty }\frac{T_{\mathrm{Ein}}^{2}}{T_{p}^{2}}\frac{\exp\frac{T_{\mathrm{Ein}}}{T_{p}}}{{\left(\exp\frac{T_{\mathrm{Ein}}}{T_{p}}-1\right)}^{2}} \end{array}$$

using the relation *T*_Ein_ ≈ 0.75 *T*_Debye_. Thus, experimentally obtained Debye temperature was used as an input for the model through that relation. *k*_*e*_ and *k*_*p*_ were the out‐of‐plane electronic and phononic thermal conductivities. The electron–phonon coupling denoted as *g*_e‐p_ facilitates the thermal equilibration between the heated electrons and the lattice on the time scale determined by the ratio *g*_e‐p_/*C*_e_. The magnetization dynamics were described microscopically by spin‐flips upon an electron–phonon scattering event. The magnetization dynamics are determined by the Boltzmann rates occupations $f_{m_{s}}$ of the *S*_*z*_ component *m*_*s*_:^[^ ^48^ ^]^

(9) $$\begin{array}{ccc} \frac{dm}{dt} & = & -\frac{1}{S}\sum_{ms=-S}^{ms=+S}m_{s}\frac{df_{m_{s}}}{dt} \end{array}$$

(10) $$\begin{array}{ccc} \frac{df_{m_{s}}}{dt} & = & -\left(W_{m_{s}}^{+}+W_{m_{s}}^{-}\right)f_{m_{s}}+W_{m_{s-1}}^{+}f_{m_{s-1}}+W_{m_{s+1}}^{-}f_{m_{s+1}} \end{array}$$

(11) $$\begin{array}{ccc} W_{m_{s}}^{\pm } & = & R\frac{Jm}{4Sk_{B}T_{c}}\frac{T_{p}}{T_{c}}\frac{e^{\mp \frac{Jm}{2Sk_{B}T_{e}}}}{\sinh(\frac{Jm}{2Sk_{B}T_{e}})}\left(S\left(S+1\right)-m_{s}\left(m_{s}\pm 1\right)\right) \end{array}$$

The rate parameter is defined as:

(12) $$\begin{array}{c} R=8\frac{a_{\mathrm{sf}}g_{e-p}T_{C}^{2}V_{\mathrm{at}}}{\mu_{\mathrm{at}}k_{B}T_{\mathrm{Debye}}^{2}} \end{array}$$

which depends on the microscopic parameters of the system, and it was proportional to the spin‐flip probability, *a*_sf_. The first term in Equation (10) corresponds to the reduction of occupation through scattering into higher and lower neighboring spin levels. The second and third terms correspond to an increase of the occupation due to scattering from lower and higher spin levels, respectively. The detailed electronic structure of the band structure of a given material was taken implicitly in terms of the parameters (e.g., electron–phonon coupling, thermal conductivity, specific heat, etc.) considered in the simulations. These were the main ingredients responsible for the magnetization dynamics at the nanosecond‐timescale investigated in the systems. The specific band structure and the nature of its bandgap however would be important at a much earlier time‐domain in the sub‐picosecond regime, where specific excitations might play a role in determining, for instance, charge transfer (a few hundreds of femtoseconds), electron‐hole pairs, and recombination processes.^[^ ^49^ ^]^ Similar arguments also apply for the generation of additional effects, such as super‐diffusive spin currents^[^ ^50^ ^]^ and laser‐induced electron currents^[^ ^51^ ^]^ which occurred at much shorter time‐scale. Although opto‐magnetic phenomena^[^ ^52^ ^]^ and optically‐induced magnetization doping^[^ ^53^ ^]^ had not been explicitly considered in the modeling, such behaviors were worth exploring in light of the results discussed here.

## Conflict of Interest

The authors declare no conflict of interest.

## Author Contributions

E.J.G.S. conceived the idea and directed the project. S.H., T.G., carried out the theoretical calculations and analyzed the data under the supervision of E.J.G.S. and U.A. S.H. and E.J.G.S. prepared the figures with inputs from T.G. and U.A. E.J.G.S. wrote the paper with an initial draft prepared by S.H. All authors discussed the results, contributed to the manuscript, and agreed on the contents included.

## Supporting information

Supporting Information

## Data Availability

The data that support the findings of this study are available in the supplementary material of this article.
