# Supplementary material for: All‐Heat Control of Magnetization Dynamics on Van der Waals Magnets
Source: Adv Mater. 2025 Jun 23;37(38):2501043. doi: 10.1002/adma.202501043 (PMC12464649; doi:10.1002/adma.202501043)
Supplement: Supplementary file 1 — Supporting Information [file ADMA-37-2501043-s001.pdf]

ADVANCED MATERIALS

Supporting Information

for *Adv. Mater.*, DOI 10.1002/adma.202501043

All-Heat Control of Magnetization Dynamics on Van der Waals Magnets

*Sumit Haldar, Theodor Griepe, Unai Atxitia and Elton J. G. Santos**

Supplementary Information for:

All-heat control of magnetization dynamics on van der Waals magnets

Sumit Haldar[1], Theodor Griepe[2], Unai Atxitia[2], and Elton J. G. Santos[1,3,4†]

[1] *Institute for Condensed Matter Physics and Complex Systems, School of Physics and Astronomy, The University of Edinburgh, EH9 3FD, United Kingdom*

[2] *Instituto de Ciencia de Materiales de Madrid, CSIC, Cantoblanco, 28049 Madrid, Spain*

[3] *Higgs Centre for Theoretical Physics, University of Edinburgh, Edinburgh UK*

[4] *Donostia International Physics Center (DIPC), 20018 Donostia-San Sebastián, Basque Country, Spain*

[†] *Corresponding author: esantos@ed.ac.uk*

This Supplementary Information file includes:

- Input parameters for the microscopic three-temperature model (M3TM) used for CGT, CrI_3, and FGT (Table S1)
- Input parameters for all considered substrates (Table S2)
- Magnetization dynamics for all 2D magnets and substrates at 0.5 mJ cm^{-2} (Figure S1)
- Fluence dependence of magnetization dynamics (Figure S2)
- Electronic temperature dynamics for CGT, CrI_3 and FGT at 14 nm and 90 nm thickness (Figure S3)
- Phonon temperature maps for 14 nm thick CGT withing hBN/CGT/substrates (Figures S4-S5)
- Phonon temperature maps for 90 nm thick CGT within hBN/CGT/substrates (Figures S6-S7)
- Phonon temperature maps for 14 nm thick CrI_3 within hBN/CrI_3/substrates (Figures S8-S9)
- Phonon temperature maps for 90 nm thick CrI_3 within hBN/CrI_3/substrates (Figures S10-S11)
- Phonon temperature maps for 14 nm thick FGT within hBN/FGT/substrates (Figures S12-S13)
- Phonon temperature maps for 90 nm thick FGT within hBN/FGT/substrates (Figures S14-S15)

- Extracted timescale constants (τ_0, τ_e, τ_m, $\tau_{m,re}$) for CGT, CrI_3 and FGT at 14 nm thickness across all substrates (Table S3).
- Fitting coefficients constants (A_1, A_2, A_3) and Root-mean-square estimation (RMSE) for CGT, CrI_3 and FGT at 14 nm thickness across all substrates (Table S4)
- Extracted timescale constants (τ_0, τ_e, τ_m, $\tau_{m,re}$) for CGT, CrI_3 and FGT at 90 nm thickness across all substrates (Table S5).
- Fitting coefficients constants (A_1, A_2, A_3) and Root-mean-square estimation (RMSE) for CGT, CrI_3 and FGT at 90 nm thickness across all substrates (Table S6)
- Power law fitting coefficients for Figure 3 in the main text (Table S7)
- Fitting of magnetization dynamics using the analytical solution of the 3TM model for 14 nm CGT across all substrates (Figures S16-S17)
- Fitting of magnetization dynamics using the analytical solution of the 3TM model for 90 nm CGT across all substrates (Figures S18-S19)
- Fitting of magnetization dynamics using the analytical solution of the 3TM model for 14 nm CrI_3 across all substrates (Figures S20-S21)
- Fitting of magnetization dynamics using the analytical solution of the 3TM model for 90 nm CrI_3 across all substrates (Figures S22-S23)
- Fitting of magnetization dynamics using the analytical solution of the 3TM model for 14 nm FGT across all substrates (Figures S24-S25)

- Fitting of magnetization dynamics using the analytical solution of the 3TM model for 90 nm FGT across all substrates (Figures S26-S27)
- Non-thermal nature of spin dynamics for 90 nm thick CGT (Figure S28)
- On the normalization on dm/dt
- Connection between magnetization dynamics and electromagnetic field stimulation
- Light absorption and heat profile differences
- Green's function analysis of the timescales of in-plane and out-of-plane heat diffusion
- Comparison of model parameters to experimental ultrafast magnetization dynamics
- Supplementary References

1 Simulation setup: Input parameters

Symbol	Description	CGT	CrI_3	FGT	Units
γ_e	Sommerfeld constant	737.87 [1]	550.0 [2]	1561.0 [3]	J $m^{-3}K^{-2}$
$C_{p\infty}$	maximum lattice spec. heat	1.38×10^6 [4]	1.8×10^6 [5]	2.0×10^6 [6,7]	J $m^{-3}K^{-1}$
g_{e-p}	electron-phonon coupling	15×10^{16} [1]	4.0×10^{16} [2]	65×10^{16}	W $m^{-3}K^{-1}$
k_e	elec. thermal conductivity	0.0	0.0	0.25 [8]	W $(m\ K)^{-1}$
k_p	ph. thermal conductivity	1.0 [9]	1.36 [2]	0.5 [8]	W $(m\ K)^{-1}$
T_C	Curie temp.	65 [4]	61 [2]	220 [7]	K
T_{Debye}	Debye temp.	200 [4]	134 [5]	190 [6]	K
T_{Ein}	Einstein temp.	150	100.5	142.5	K
S	effective spin	3/2 [4]	3/2 [2]	2	
μ_{at}	atom. magn. moment	4.0	4 [2]	2.0 [7]	μ_B
a_{sf}	spin-flip prob.	0.05	0.175 [2]	0.04	
V_{at}	atomic volume	100	100	100	Å^3

Table S1: Input parameters for the 3TM used in the ultrafast laser-induced magnetic dynamics on CGT, CrI_3, and FGT are taken from the literature. The maximum lattice specific heat value extracted from the specific heat vs. temperature plot at the Debye temperature. Thermal conductivity is considered along the interlayer distance.

Substrate	$C_{p\infty}$<br>J $m^{-3}K^{-1}$	T_{Debye}<br>K	k_p<br>W $(m\ K)^{-1}$
WSe_2	1.98×10^6[10]	160[11]	0.35[12]
Stanene	0.8×10^6[13]	72.5[14]	0.83[15]
Al_2O_3	3.55×10^6[16]	980[17]	1.0 [18]
$MoSe_2$	1.99×10^6[19]	177.6[19]	1.2 [20]
SiO_2	1.9×10^6[21]	403[21]	1.5[22, 23]
WS_2	1.08×10^6[19]	213[19]	1.7[24]
Bi_2Te_3	1.2×10^6[25]	165[25]	1.8[26, 27]
ZnO	3.53×10^6[28]	416[28]	2.3[29]
ITO	3.5×10^6[30]	1000[30]	3.2[31]
Phosphorene	2.1×10^6[32]	278.66[32]	4.0[33]
Silicene	2.0×10^6[34]	640[35]	4.5[36]
MoS_2	2.0×10^6 [37]	280[38]	5.0[39]
hBN	2.64×10^6 [40]	400 [41]	5.0 [42, 43]
Graphene	4.68×10^6 [44]	1911 [45]	6.0[44]
Black phosphorus	2.17×10^6 [46]	370 [47]	6.5 [48]
AlN	3.53×10^6 [49]	1150 [50]	8.5 [51]

Table S2: Input parameters for various substrates used in 3TM to simulate utrafast-laser induced magnetization dynamics of CGT, CrI_3, and FGT. Thermal conductivity values are considered perpendicular to the surface.

2 Magnetization dynamics for all 2D magnets and substrates at 0.5 mJ cm^{-2}

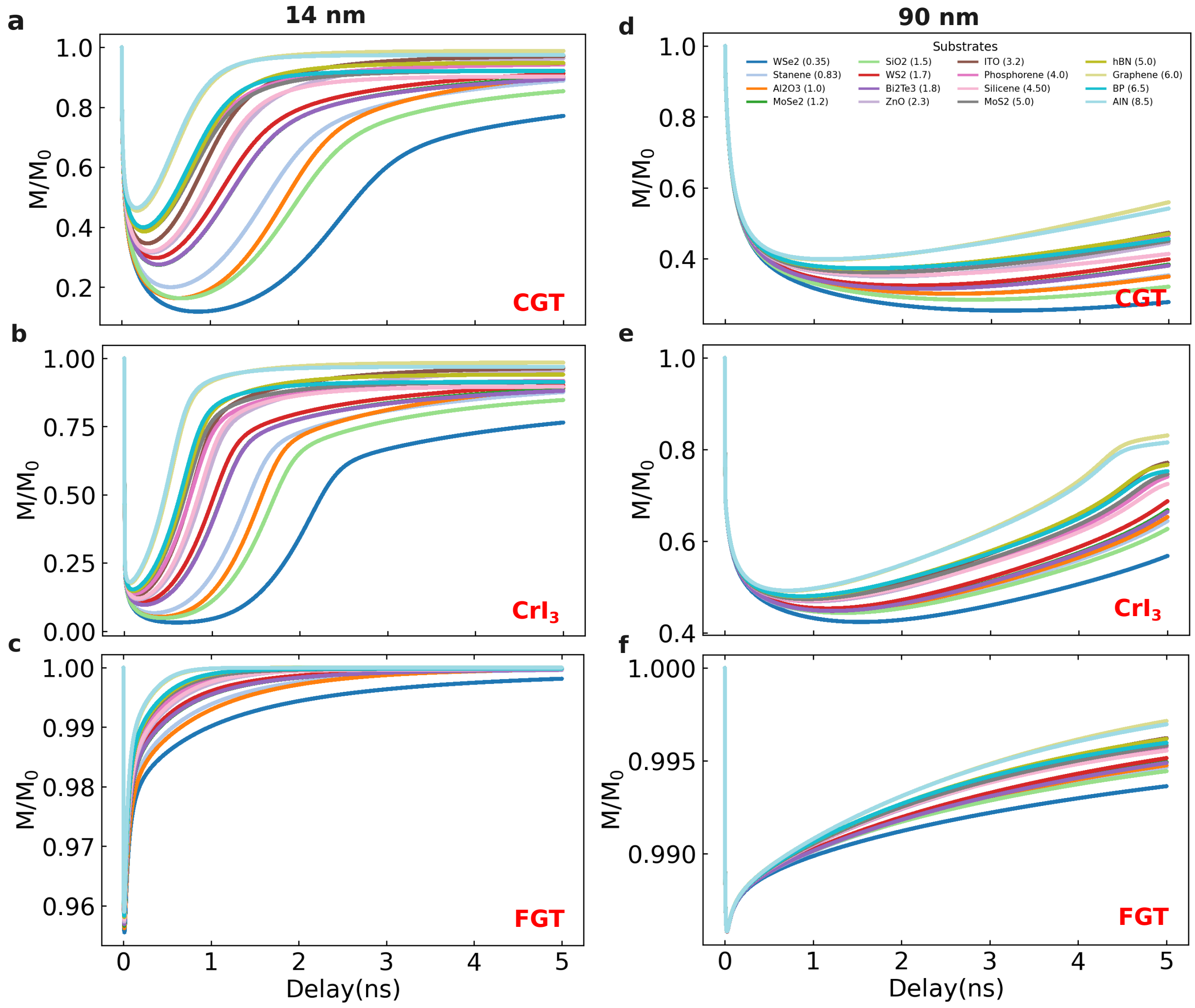

Figure S1: Normalized magnetization dynamics after the laser excitation with a fluence 0.5 mJ/cm^2 for 14 nm and 90 nm thick CGT **a-d**, CrI_3 **b-e**, and FGT **c-f**.

3 Fluence dependence of magnetization dynamics

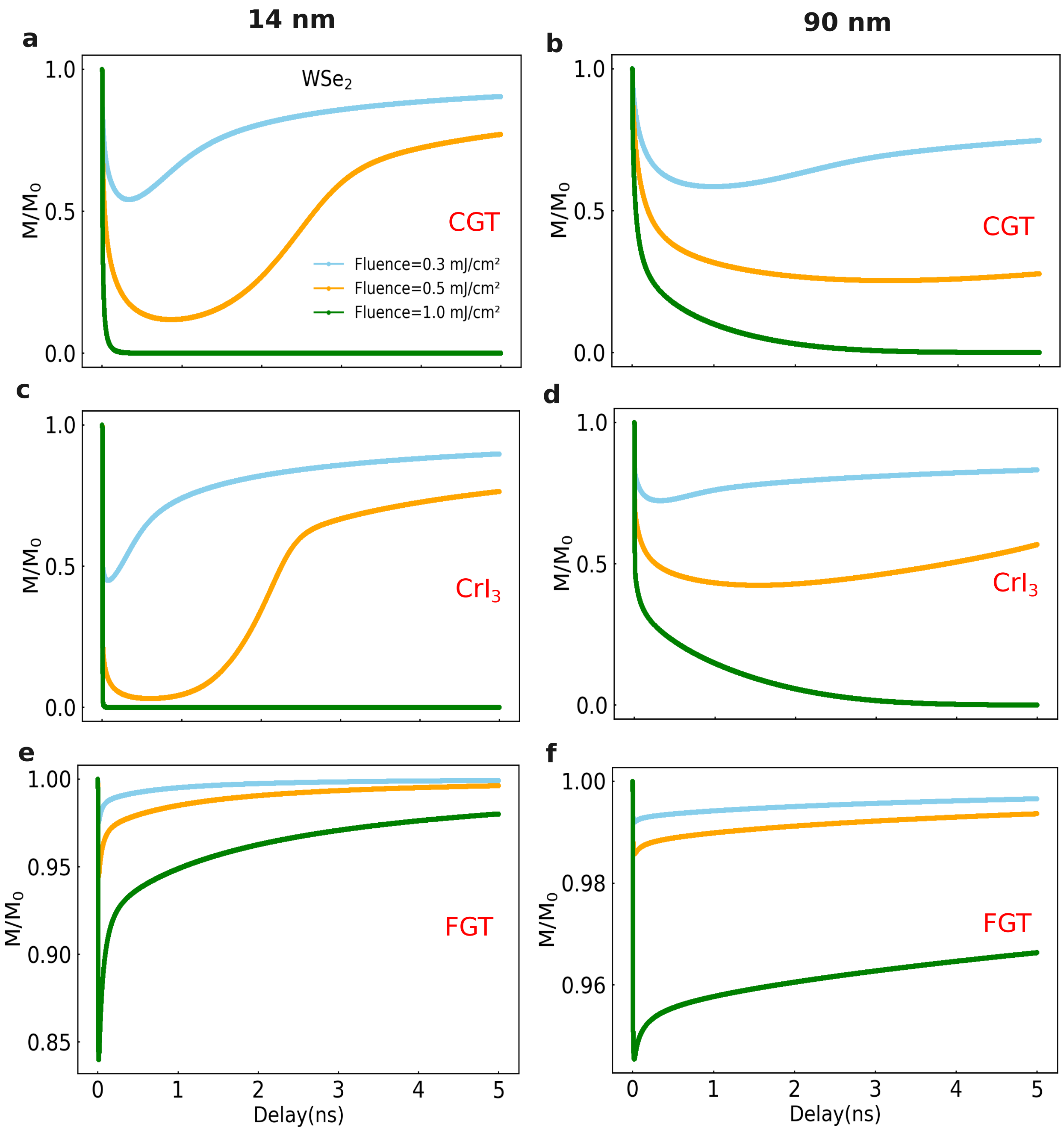

Figure S2: Fluence dependence of normalized magnetization dynamics for 14 nm and 90 nm thick CGT **a-b**, CrI_3 **c-d**, and FGT **e-f**.

4 Electronic temperature dynamics for CGT, CrI_3, and FGT at 14 nm and 90 nm thickness

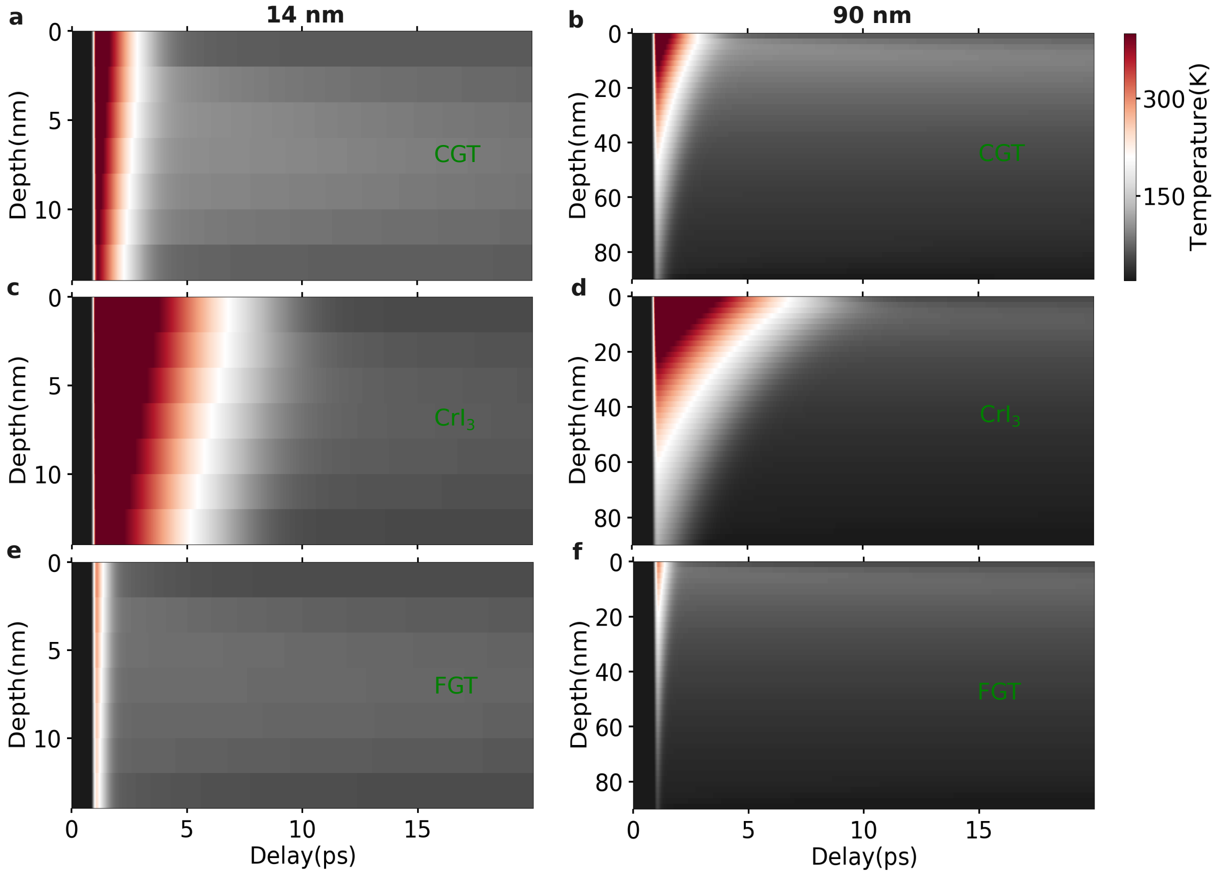

Figure S3: Ultrafast laser-induced electronic temperature dynamics across the hBN/vdW/substrate heterostructure. The hBN layer has a thickness of 14 nm, while the SiO_2 substrate is 300 nm. The thickness of the vdW material layers is 14 nm and 90 nm for CGT in panels **a** and **d**, CrI_3 in panels **b** and **e**, and FGT in panels **c** and **f**.

5 Phonon temperature maps

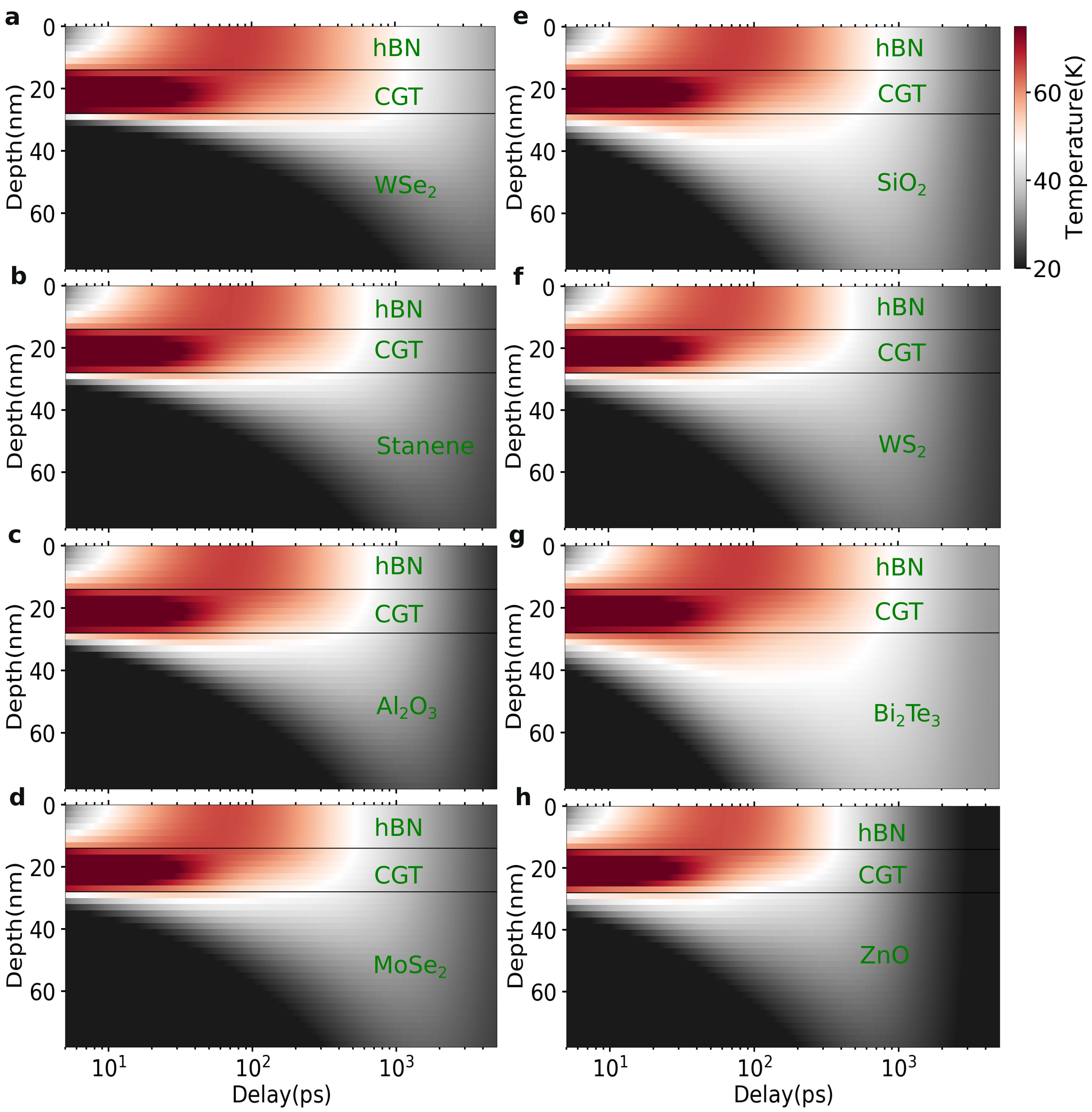

Figure S4: Phonon temperature dynamics across the thickness of the hBN/CGT/substrate heterostructure. The thicknesses of hBN and substrate are 14 nm, 300 nm, and 14 nm for CGT. **(a)** evolution of the phonon temperature with WSe_2 as the substrate, **(b)** with Stanene, **(c)** with Al_2O_3, **(d)** with $MoSe_2$, **(e)** with SiO_2, **(f)** with WS_2, **(g)** with Bi_2Te_3, **(h)** with ZnO as the substrate.

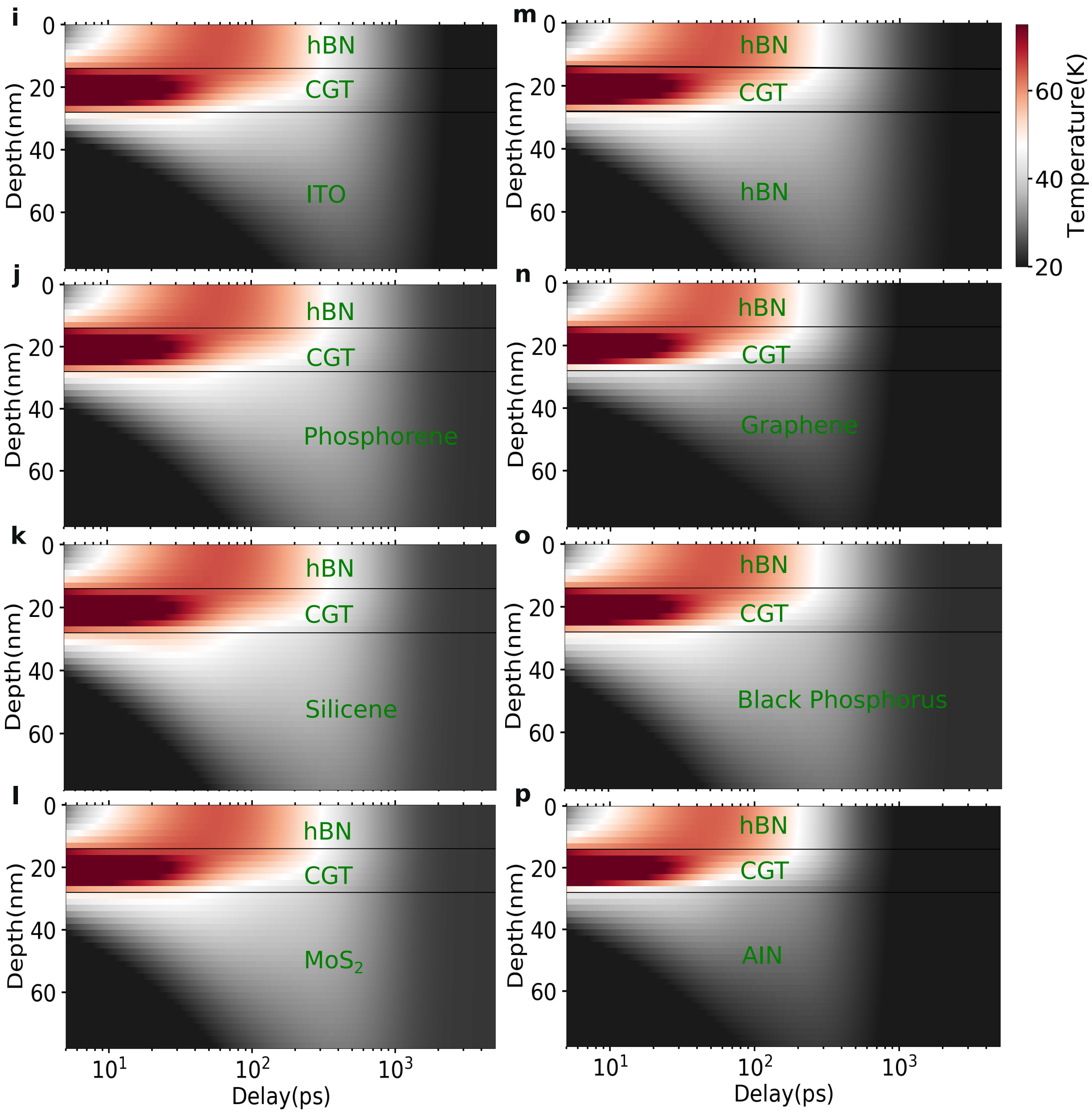

Figure S5: Phonon temperature dynamics across the thickness of the hBN/CGT/substrate heterostructure. The thicknesses of hBN and substrate are 14 nm, 300 nm, and 14 nm for CGT. **(i)** evolution of the phonon temperature with INO as the substrate, **(j)** with Phosphorence, **(k)** with Silicene, **(l)** with MoS_2, **(m)** with hBN, **(n)** with Graphene, **(o)** with Black Phosphorus, **(p)** with AIN as the substrate.

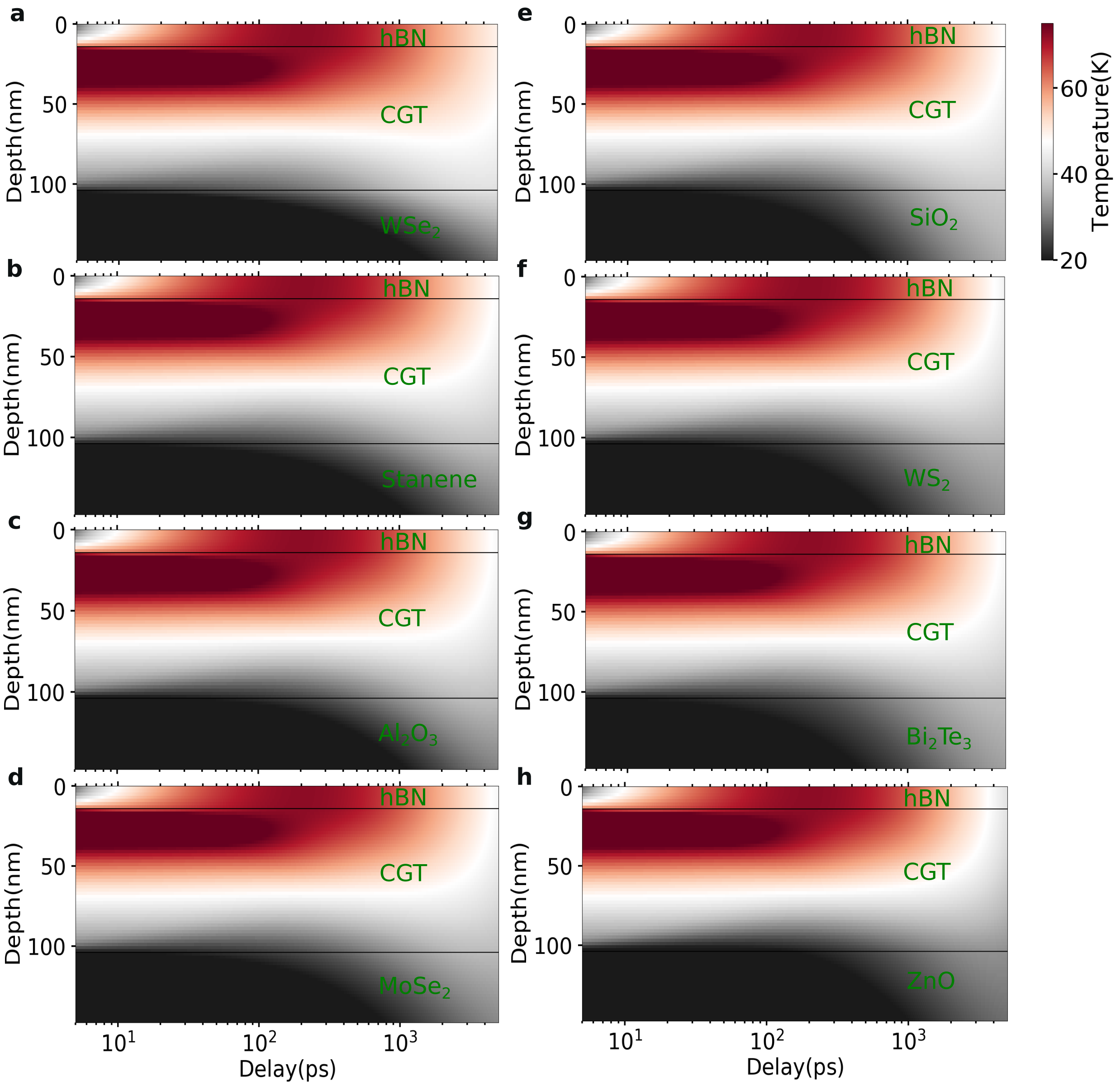

Figure S6: Phonon temperature dynamics across the thickness of the hBN/CGT/substrate heterostructure. The thicknesses of hBN and substrate are 14 nm, 300 nm, and 90 nm for CGT. **(a)** evolution of the phonon temperature with WSe_2 as the substrate, **(b)** with Stanene, **(c)** with Al_2O_3, **(d)** with $MoSe_2$, **(e)** with SiO_2, **(f)** with WS_2, **(g)** with Bi_2Te_3, **(h)** with ZnO as the substrate.

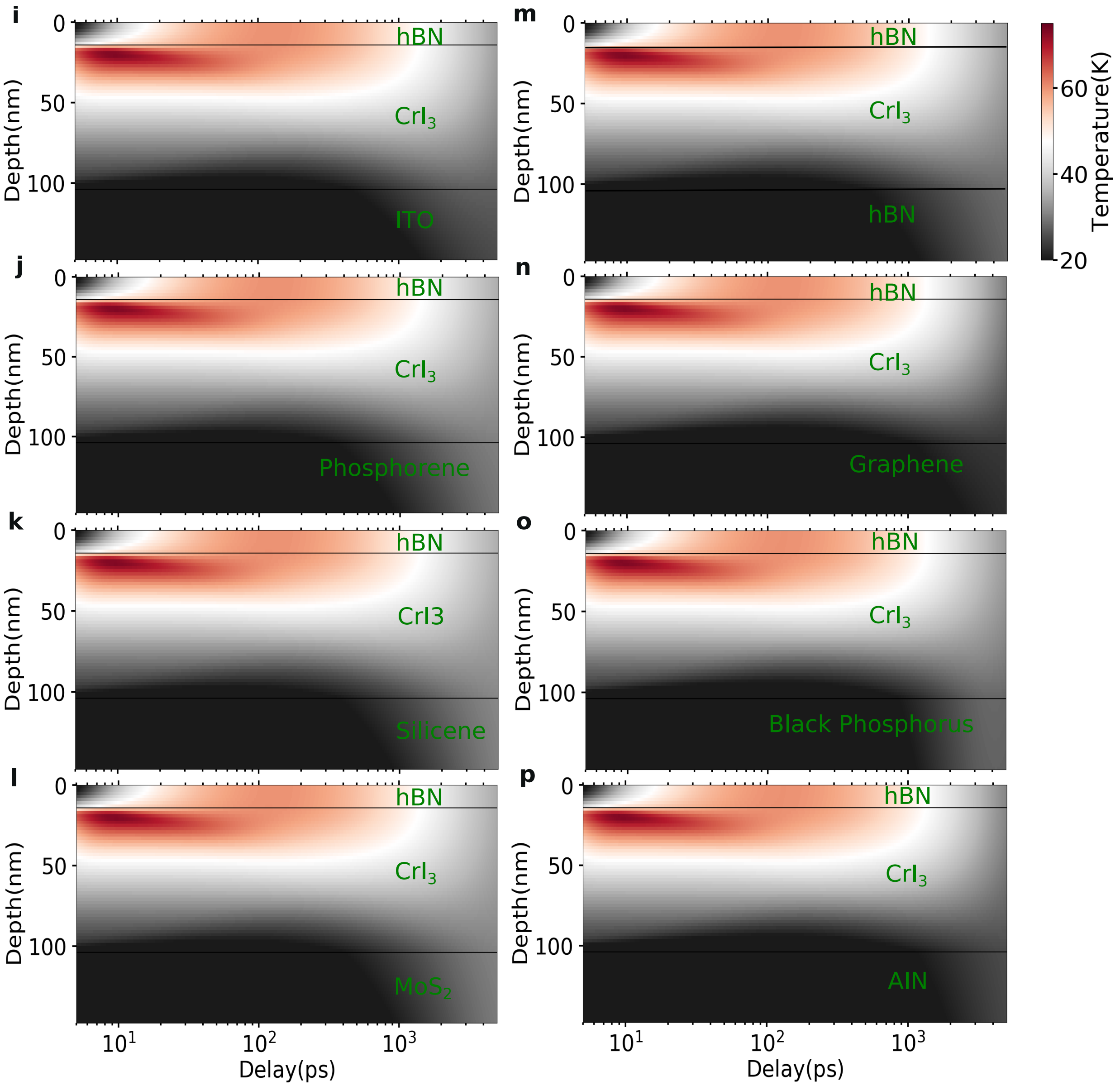

Figure S7: Phonon temperature dynamics across the thickness of the hBN/CGT/substrate heterostructure. The thicknesses of hBN and substrate are 14 nm, 300 nm, and 90 nm for CGT. **(i)** evolution of the phonon temperature with INO as the substrate, **(j)** with Phosphorence, **(k)** with Silicene, **(l)** with MoS_2, **(m)** with hBN, **(n)** with Graphene, **(o)** with Black Phosphorus, **(p)** with AIN as the substrate.

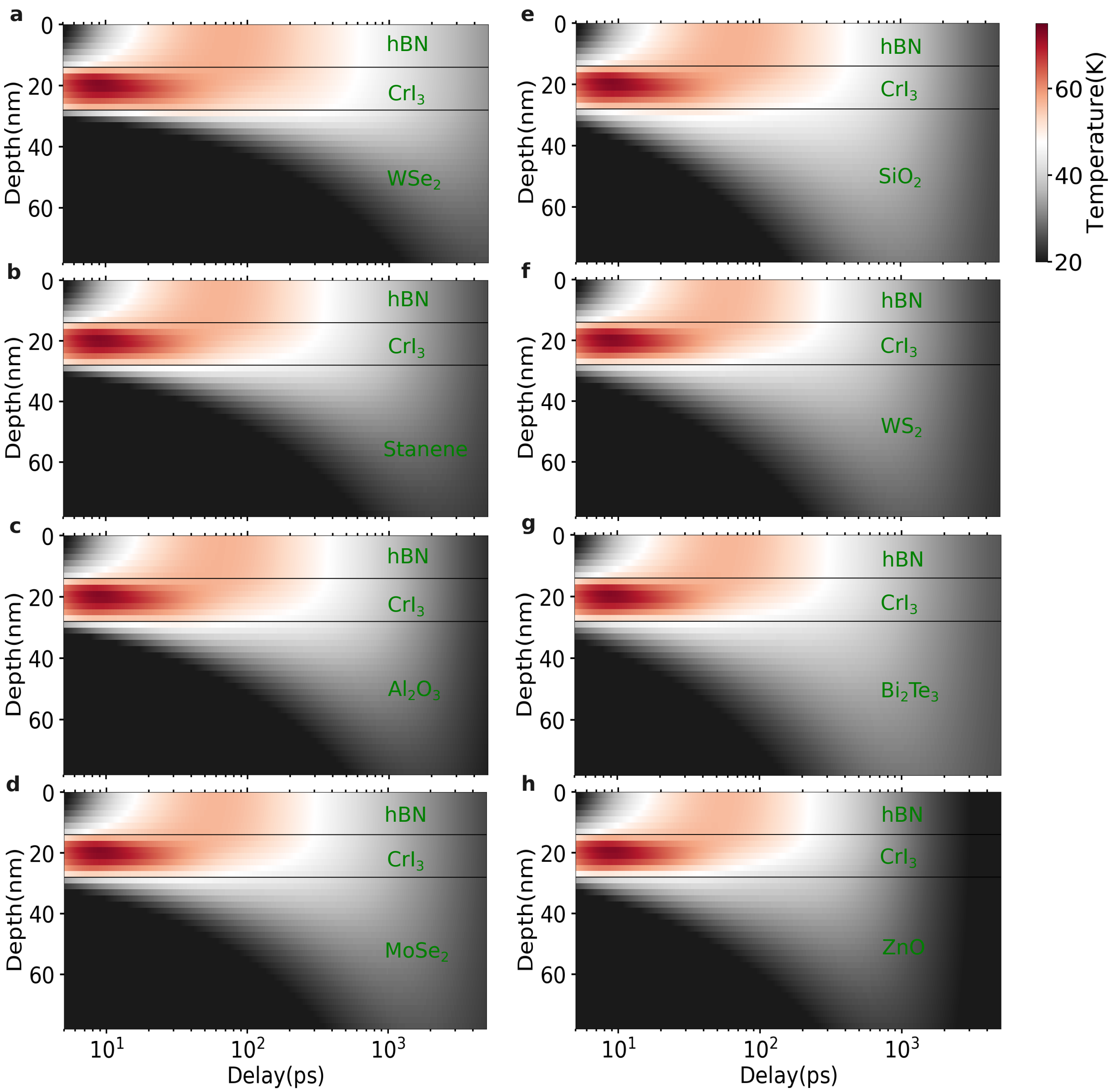

Figure S8: Phonon temperature dynamics across the thickness of the hBN/CGT/substrate heterostructure. The thicknesses of hBN and substrate are 14 nm, 300 nm, and 14 nm for CrI_3. **(a)** evolution of the phonon temperature with WSe_2 as the substrate, **(b)** with Stanene, **(c)** with Al_2O_3, **(d)** with $MoSe_2$, **(e)** with SiO_2, **(f)** with WS_2, **(g)** with Bi_2Te_3, **(h)** with ZnO as the substrate.

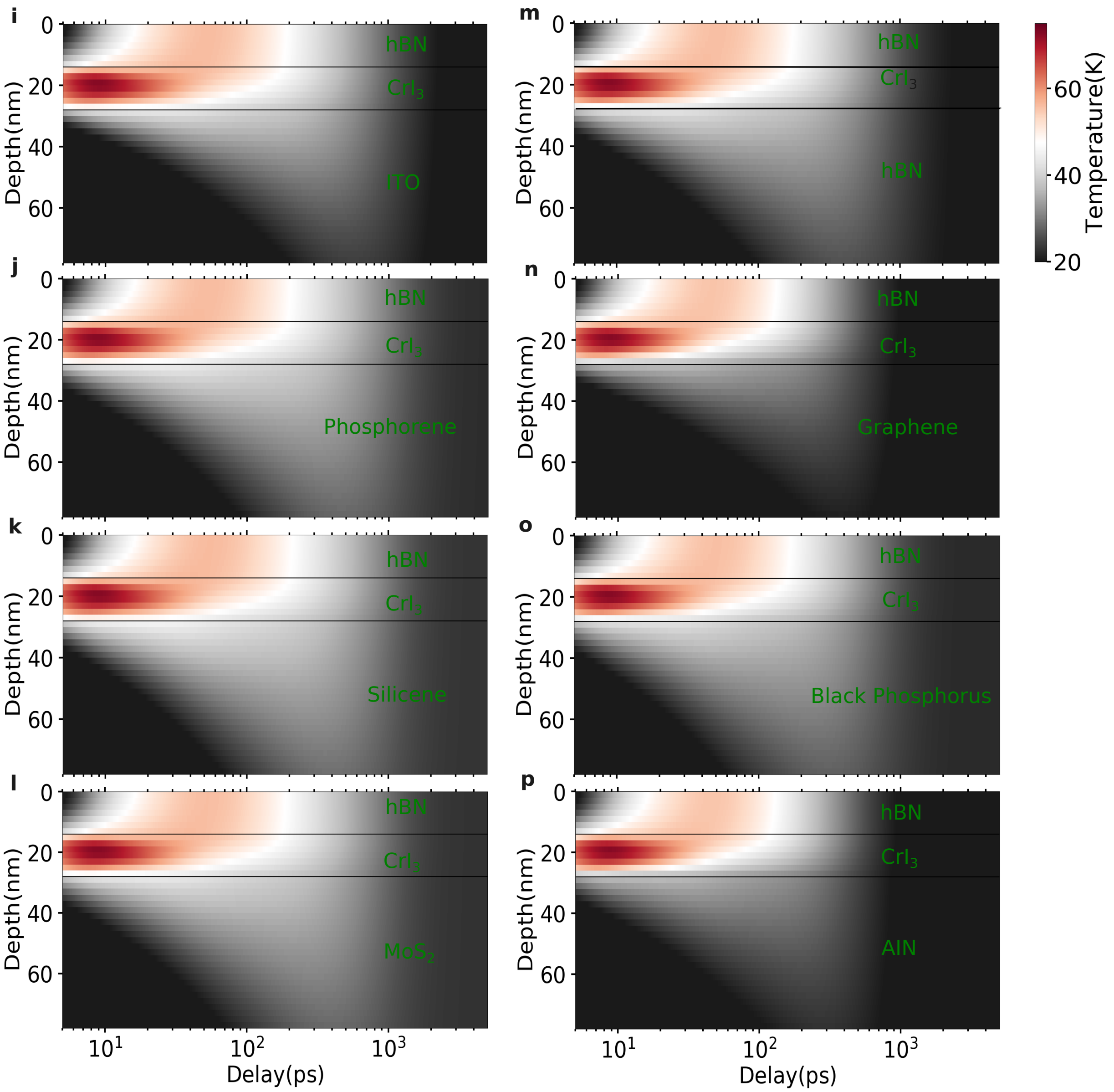

Figure S9: Phonon temperature dynamics across the thickness of the hBN/CGT/substrate heterostructure. The thicknesses of hBN and substrate are 14 nm, 300 nm, and 14 nm for CrI_3. **(i)** evolution of the phonon temperature with INO as the substrate, **(j)** with Phosphorence, **(k)** with Silicene, **(l)** with MoS_2, **(m)** with hBN, **(n)** with Graphene, **(o)** with Black Phosphorus, **(p)** with AIN as the substrate.

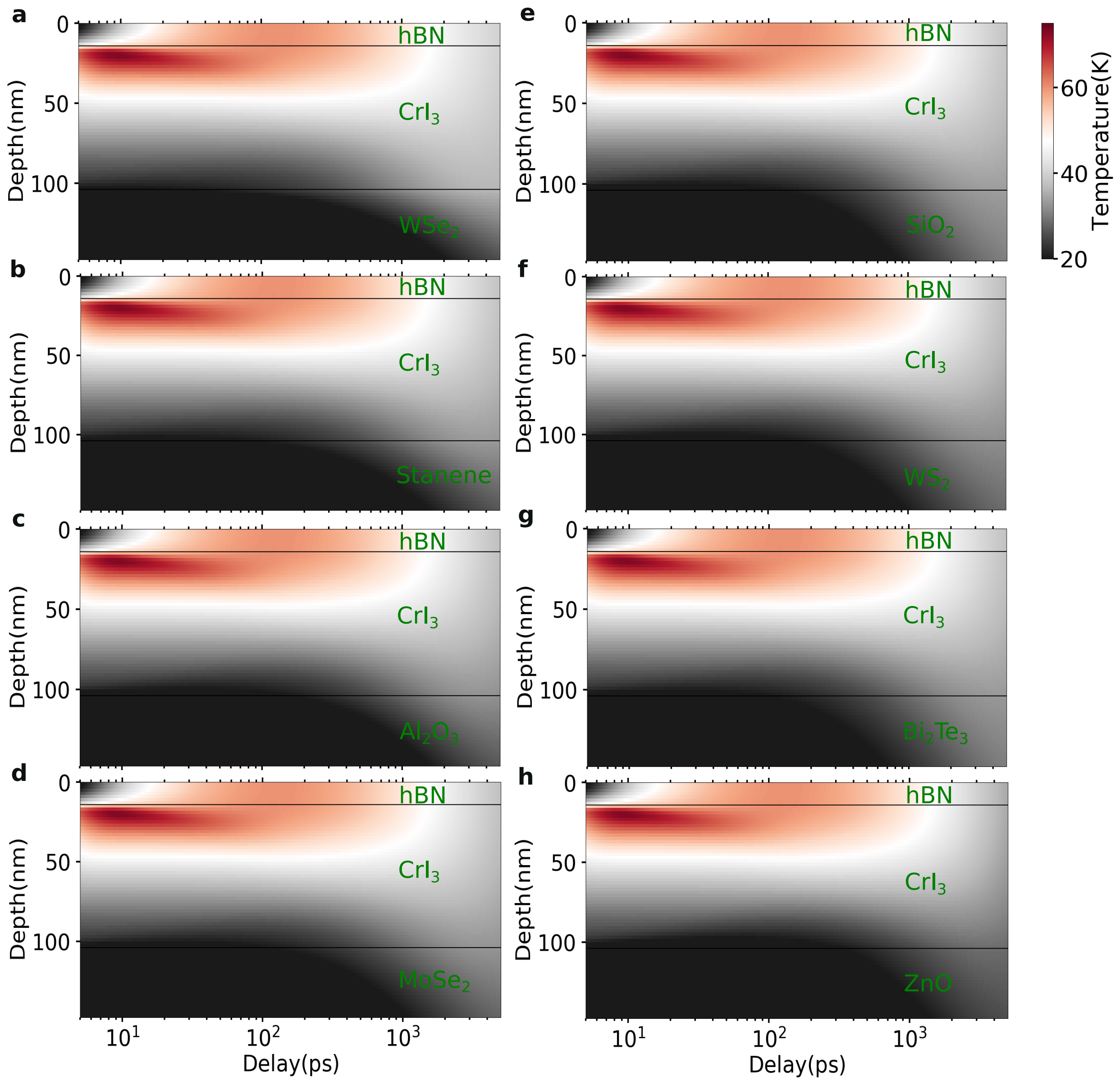

Figure S10: Phonon temperature dynamics across the thickness of the hBN/CGT/substrate heterostructure. The thicknesses of hBN and substrate are 14 nm, 300 nm, and 90 nm for CrI_3. **(a)** evolution of the phonon temperature with WSe_2 as the substrate, **(b)** with Stanene, **(c)** with Al_2O_3, **(d)** with $MoSe_2$, **(e)** with SiO_2, **(f)** with WS_2, **(g)** with Bi_2Te_3, **(h)** with ZnO as the substrate.

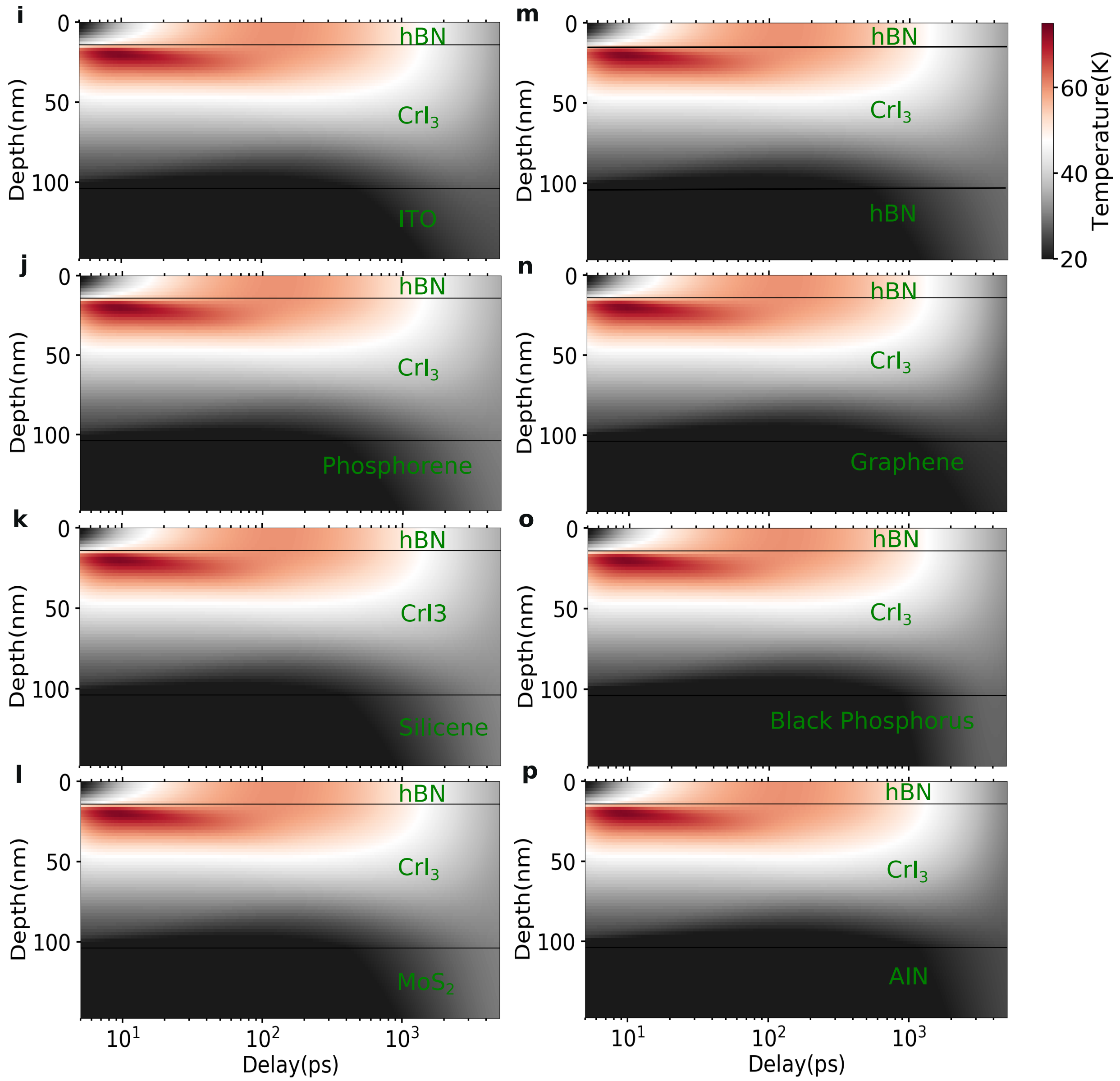

Figure S11: Phonon temperature dynamics across the thickness of the hBN/CGT/substrate heterostructure. The thicknesses of hBN and substrate are 14 nm, 300 nm, and 90 nm for CrI_3. **(i)** evolution of the phonon temperature with INO as the substrate, **(j)** with Phosphorence, **(k)** with Silicene, **(l)** with MoS_2, **(m)** with hBN, **(n)** with Graphene, **(o)** with Black Phosphorus, **(p)** with AIN as the substrate.

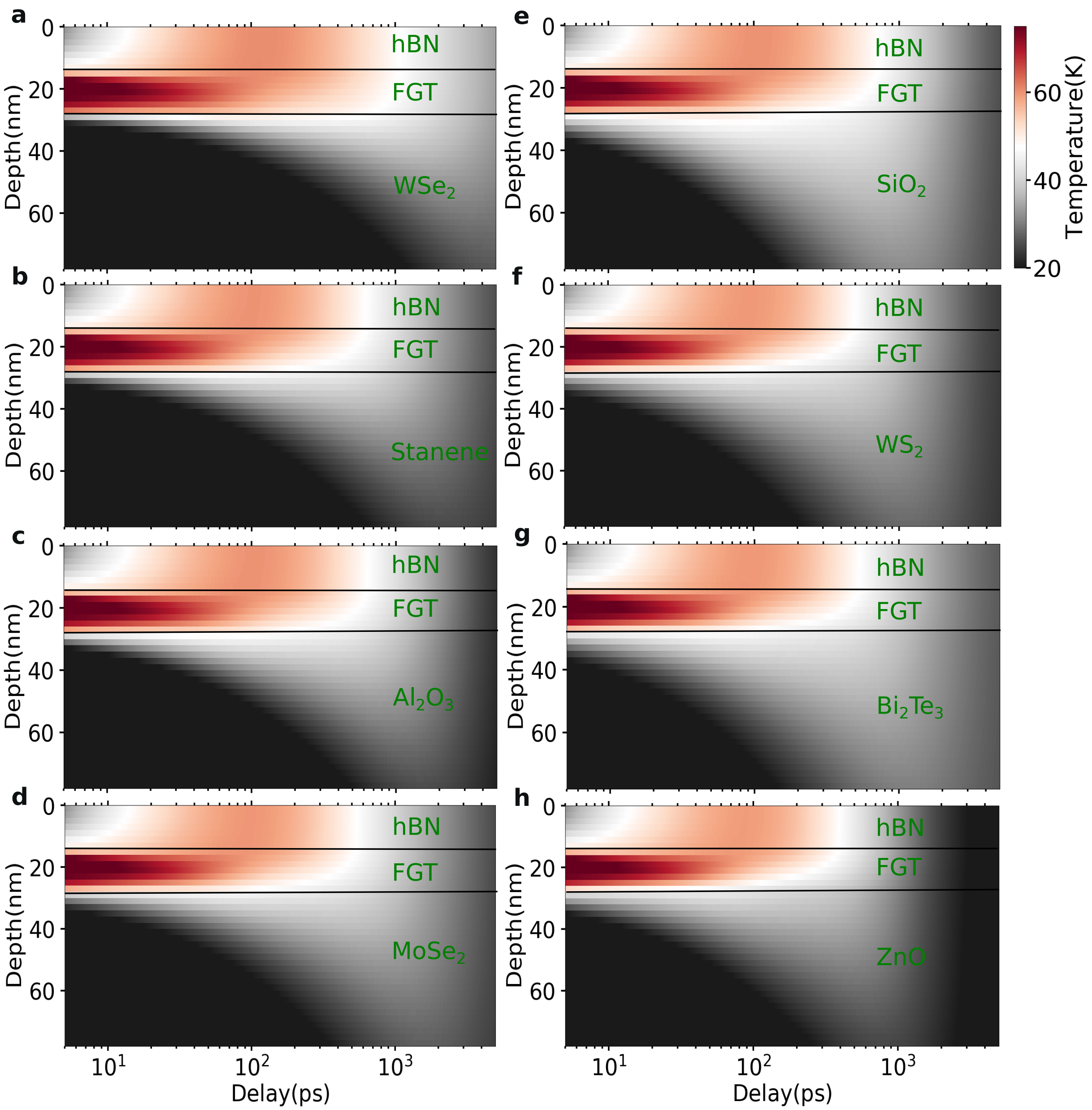

Figure S12: Phonon temperature dynamics across the thickness of the hBN/CGT/substrate heterostructure. The thicknesses of hBN and substrate are 14 nm, 300 nm, and 14 nm for FGT. **(a)** evolution of the phonon temperature with WSe_2 as the substrate, **(b)** with Stanene, **(c)** with Al_2O_3, **(d)** with $MoSe_2$, **(e)** with SiO_2, **(f)** with WS_2, **(g)** with Bi_2Te_3, **(h)** with ZnO as the substrate.

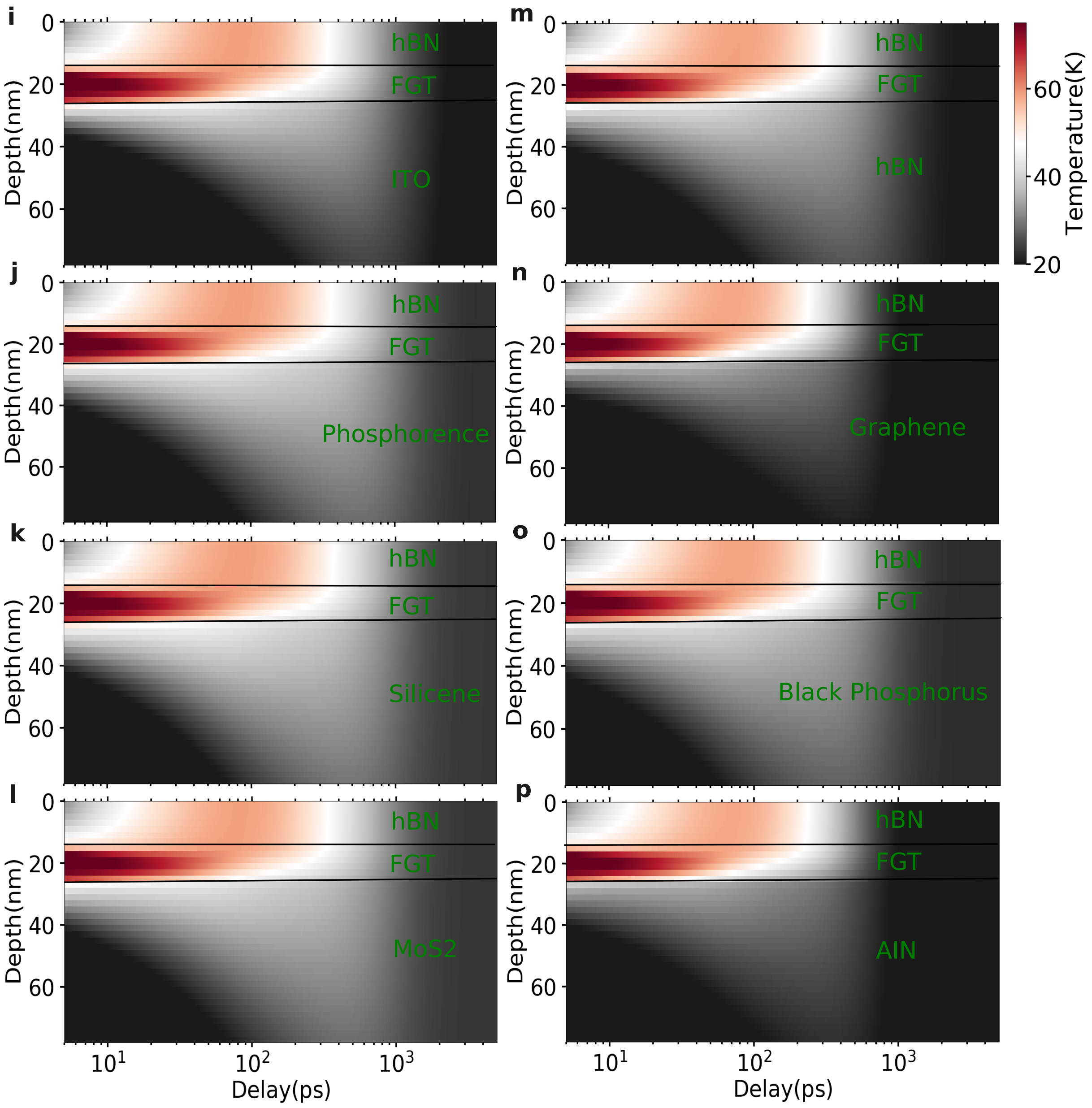

Figure S13: Phonon temperature dynamics across the thickness of the hBN/CGT/substrate heterostructure. The thicknesses of hBN and substrate are 14 nm, 300 nm, and 14 nm for FGT. **(i)** evolution of the phonon temperature with INO as the substrate, **(j)** with Phosphorence, **(k)** with Silicene, **(l)** with MoS_2, **(m)** with hBN, **(n)** with Graphene, **(o)** with Black Phosphorus, **(p)** with AIN as the substrate.

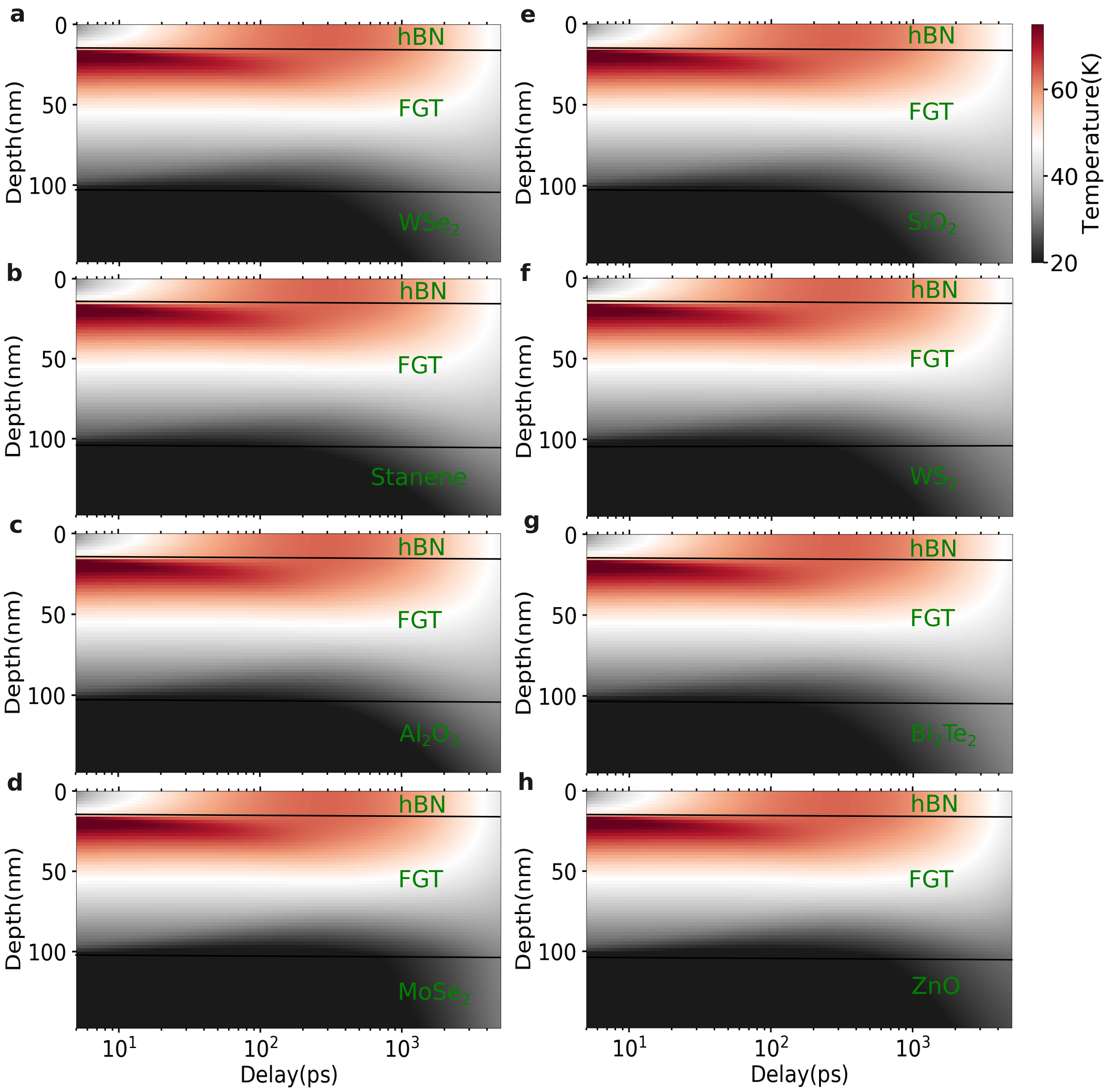

Figure S14: Phonon temperature dynamics across the thickness of the hBN/CGT/substrate heterostructure. The thicknesses of hBN and substrate are 14 nm, 300 nm, and 90 nm for FGT. **(a)** evolution of the phonon temperature with WSe_2 as the substrate, **(b)** with Stanene, **(c)** with Al_2O_3, **(d)** with $MoSe_2$, **(e)** with SiO_2, **(f)** with WS_2, **(g)** with Bi_2Te_3, **(h)** with ZnO as the substrate.

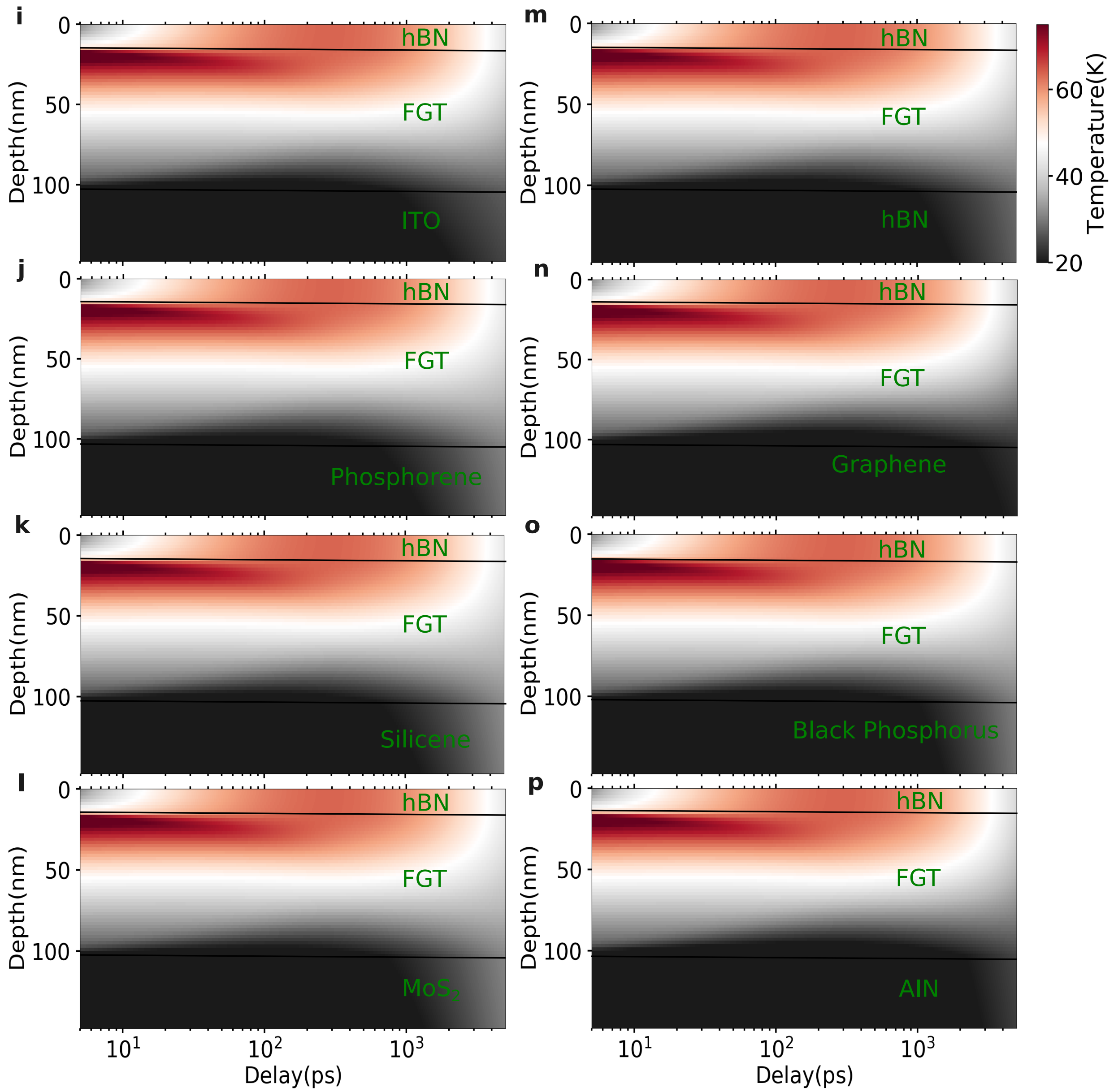

Figure S15: Phonon temperature dynamics across the thickness of the hBN/CGT/substrate heterostructure. The thicknesses of hBN and substrate are 14 nm, 300 nm, and 90 nm for FGT. **(i)** evolution of the phonon temperature with INO as the substrate, **(j)** with Phosphorence, **(k)** with Silicene, **(l)** with MoS_2, **(m)** with hBN, **(n)** with Graphene, **(o)** with Black Phosphorus, **(p)** with AIN as the substrate.

6 Fitting coefficients

Substrate	CGT				CrI_3				FGT			
	τ_0 (ns)	τ_e (ps)	τ_m (ps)	$\tau_{m,re}$ (ns)	τ_0 (ns)	τ_e (ps)	τ_m (ps)	$\tau_{m,re}$ (ns)	τ_0 (ns)	τ_e (ps)	τ_m (ps)	$\tau_{m,re}$ (ns)
WSe_2	0.4913	2.7994	191.97	5.2047	0.3084	5.115	77.94	4.612	0.4131	0.4432	2.6411	0.9181
Stanene	0.2461	3.8059	146.65	4.4798	0.1298	3.7396	84.57	0.5924	0.0155	0.1728	2.5515	0.6547
Al_2O_3	0.2745	2.8953	155.19	6.3990	0.4866	4.1323	58.38	3.9102	0.0118	0.1916	2.9894	0.6017
$MoSe_2$	0.2227	4.3129	133.69	3.6928	0.1292	3.6145	79.34	0.6258	0.0154	0.1562	2.3770	0.5819
SiO_2	0.2983	3.3829	167.63	6.2656	0.5666	3.9590	60.24	3.4265	0.0140	2.8232	0.1927	0.7433
WS_2	0.2174	3.7442	128.58	4.3269	0.3099	4.4508	62.55	2.1003	0.0125	0.1585	2.5647	0.4841
Bi_2Te_3	0.3882	2.2813	182.95	2.8670	0.1864	3.5520	84.30	0.8684	0.0165	0.1536	2.1954	0.5895
ZnO	0.2086	1.8762	113.85	3.7591	0.2122	4.2785	60.62	3.0836	0.0076	0.1858	2.8284	0.2734
ITO	0.1810	1.7397	100.95	3.2449	0.0741	5.2462	60.25	2.5189	0.0065	0.2044	2.8070	0.2029
Phosphorene	0.2350	1.5159	110.86	2.6738	0.1580	3.9394	66.47	1.7969	0.0078	0.1778	2.6897	0.2489
Silicene	0.2427	1.2615	112.70	2.4774	0.1223	3.8133	69.99	1.5763	0.0077	0.1894	2.7663	0.2470
MoS_2	0.2248	1.3604	105.02	2.3221	0.1046	3.8508	64.60	1.4165	0.0073	0.1878	2.6770	0.2116
hBN	0.1795	1.5145	96.22	2.6164	0.0610	4.9448	55.76	1.7770	0.0061	0.2156	2.7969	0.1735
Graphene	0.1117	1.9576	72.25	2.6429	0.0155	5.714	36.51	1.9504	0.004	0.2679	3.1256	0.1016
Black phosphorus	0.1836	1.3290	94.56	2.1390	0.0677	4.062	55.84	1.3295	0.0062	0.2193	2.7966	0.1633
AlN	0.1130	1.8298	71.34	2.3542	0.0217	4.058	37.85	1.6071	0.0038	0.2707	3.1442	0.1633

Table S3: Extracted timescales of rapid demagnetization (τ_e), slower demagnetization (τ_m), remagnetization ($\tau_{m,re}$), and heat diffusion to the substrate (τ_0) obtained by fitting the analytical solution of the 3TM for 14 nm thick CGT, CrI_3, and FGT samples across all substrates.

Substrate	CGT				CrI_3				FGT			
	A_1	A_2	A_3	RMSE	A_1	A_2	A_3	RMSE	A_1	A_2	A_3	RMSE
WSe_2	1.112	12.01	0.058	0.0038	0.972	18.584	0.235	0.0027	0.014	4.027	-0.009	0.00016
Stanene	1.381	12.31	0.079	0.0039	1.778	17.761	0.192	0.0019	0.043	0.440	-0.008	0.00012
Al_2O_3	1.468	18.40	0.117	0.0040	0.845	9.321	0.137	0.0059	0.042	0.452	-0.010	0.00013
$MoSe_2$	1.259	9.361	0.061	0.0048	1.696	17.140	0.199	0.0018	0.044	0.471	-0.007	0.00014
SiO_2	1.443	16.65	0.099	0.0031	0.848	10.212	0.151	0.0052	0.041	0.029	-0.009	0.00011
WS_2	1.246	10.40	0.073	0.0045	0.888	9.157	0.117	0.0063	0.045	0.502	-0.008	0.00014
Bi_2Te_3	1.442	30.2357	0.162	0.0031	1.362	17.573	0.195	0.0027	0.044	0.441	-0.007	0.00014
ZnO	1.341	21.00	0.163	0.0064	1.008	9.352	0.125	0.0055	0.044	0.440	-0.010	0.00017
ITO	1.321	19.91	0.169	0.0046	1.850	9.363	0.101	0.0051	0.042	0.380	-0.011	0.00018
Phosphorene	1.234	26.70	0.200	0.0046	1.226	11.868	0.156	0.0035	0.044	0.444	-0.010	0.00017
Silicene	1.356	36.55	0.253	0.0042	1.544	13.778	0.175	0.0026	0.043	0.417	-0.010	0.00016
MoS_2	1.241	29.21	0.222	0.0049	1.616	12.878	0.175	0.0027	0.043	0.407	-0.010	0.00017
hBN	1.289	22.47	0.191	0.0043	2.155	10.268	0.131	0.0034	0.041	0.351	-0.012	0.00017
Graphene	1.214	11.41	0.132	0.0045	5.343	17.060	0.106	0.0050	0.040	0.298	-0.014	0.00015
Black phosphorus	1.294	26.58	0.220	0.0041	2.102	12.061	0.180	0.0021	0.041	0.343	-0.012	0.00017
AlN	1.196	12.11	0.140	0.0039	4.056	12.475	0.130	0.0043	0.041	0.295	-0.015	0.00015

Table S4: The fitting constants A_1, A_2, and A_3 describe the normalized magnetization dynamics in 14 nm thick CGT, Cr_3, and FGT samples. A_1 corresponds to the magnetization after the system reaches temporal equilibrium between the thermal baths, while A_2 captures the initial rise in electron temperature. A_3 accounts for state filling amplitude. Root Mean Square Error (RMSE) measures the discrepancy between the simulated data and the fitted model. Heat diffusion to the substrate is modeled by the function $F(\tau_0,t)$, where for 14 nm thick samples is given by $F(\tau_0,t) = (t/\tau_0+1)^{-1}$, and for 90 nm thick samples, $F(\tau_0,t) = (t/\tau_0+2)^{-1}$. In earlier studies [3,52], the form of $F(\tau_0,t)$ has varied, taking either the form $A_1/\sqrt{t/\tau_0+1}$ or $A_1\exp(-t/\tau_0)$ which better described the corresponding data. We used the ones resulting in the best agreement with our dataset.

Substrate	CGT				CrI_3				FGT			
	τ_0 (ns)	τ_e (ps)	τ_m (ps)	$\tau_{m,re}$ (ns)	τ_0 (ns)	τ_e (ps)	τ_m (ps)	$\tau_{m,re}$ (ns)	τ_0 (ns)	τ_e (ps)	τ_m (ps)	$\tau_{m,re}$ (ns)
WSe_2	5.0428	48.1269	546.16	1.3279	12.844	4.2975	138.53	1.7057	0.8233	0.0577	1.7436	1.0511
Stanene	2.3203	47.0685	562.79	1.1708	10.508	4.3548	105.69	1.4610	0.6003	0.0575	1.9211	0.8767
Al_2O_3	2.2659	46.5723	556.96	1.1568	8.3346	4.3550	105.71	1.3835	0.5543	0.0577	1.9655	0.8105
$MoSe_2$	1.9818	46.8959	571.83	1.1462	8.6832	4.3680	106.65	1.4368	0.5516	0.0566	1.9534	0.8155
SiO_2	2.9412	45.8867	537.40	1.1944	11.644	4.3514	106.34	1.3834	0.6758	0.0605	1.8948	0.9708
WS_2	1.7185	46.3499	573.18	1.1164	6.9479	4.3828	108.52	1.3662	0.4907	0.0559	2.0085	0.7310
Bi_2Te_3	2.1112	46.2310	559.53	1.1465	9.3736	4.3795	109.30	1.3863	0.5910	0.0579	1.9258	0.8558
ZnO	0.7829	47.3782	617.10	0.9783	3.5120	4.4046	109.19	1.3260	0.3049	0.0507	2.2966	0.5009
ITO	0.4500	51.6967	613.37	0.8780	2.6941	4.4244	111.41	1.3126	0.2530	0.0489	2.4573	0.4352
Phosphorene	1.1878	42.9901	546.26	1.0111	3.9348	4.4896	126.17	1.0533	0.3506	0.0533	2.1712	0.5394
Silicene	1.4868	40.6783	502.67	1.0098	3.9771	4.5458	135.84	0.9324	0.3760	0.0549	2.1204	0.5563
MoS_2	1.2000	41.8518	528.17	0.9943	3.6390	4.5379	135.21	0.9382	0.3459	0.0537	2.1713	0.5260
hBN	0.6246	44.2099	575.04	0.8832	2.7305	4.5032	126.97	1.0600	0.2569	0.0495	2.4135	0.4325
Graphene	0.2917	57.4153	571.63	1.1207	1.2110	4.4381	114.30	1.5432	0.1650	0.0453	3.0061	0.3175
Black phosphorus	1.0966	40.0978	507.91	0.9562	2.2628	4.5738	131.15	0.7559	0.2972	0.0520	2.2642	0.4678
AlN	0.2755	58.5338	563.49	0.8855	1.5269	4.5111	107.46	1.1459	0.1739	0.0459	2.8854	0.3266

Table S5: Extracted timescales of rapid demagnetization (τ_e), slower demagnetization (τ_m), remagnetization ($\tau_{m,re}$), and heat diffusion to the substrate (τ_0) obtained by fitting the analytical solution of the 3TM for 90 nm thick CGT, CrI_3, and FGT samples across all substrates.

Substrate	CGT				CrI_3				FGT			
	A_1	A_2	A_3	RMSE	A_1	A_2	A_3	RMSE	A_1	A_2	A_3	RMSE
WSe_2	0.721	2.952	-0.363	0.0030	0.365	5.044	-0.135	0.0033	0.027	0.775	0.006	0.000068
Stanene	0.800	3.139	-0.402	0.0031	0.340	5.348	-0.138	0.0031	0.027	0.769	0.006	0.000063
Al_2O_3	0.792	3.118	-0.398	0.0031	0.339	5.354	-0.138	0.0031	0.027	0.785	0.006	0.000063
$MoSe_2$	0.830	3.216	-0.417	0.0031	0.340	5.384	-0.139	0.0032	0.028	0.823	0.006	0.000063
SiO_2	0.752	3.000	-0.378	0.0032	0.340	5.395	-0.138	0.0031	0.025	0.663	0.005	0.000063
WS_2	0.853	3.260	-0.429	0.0031	0.341	5.469	-0.139	0.0031	0.029	0.881	0.007	0.000064
Bi_2Te_3	0.813	3.156	-0.409	0.0032	0.342	5.516	-0.139	0.0031	0.027	0.758	0.006	0.000063
ZnO	1.164	3.761	-0.585	0.0029	0.342	5.473	-0.140	0.0030	0.035	1.373	0.009	0.000094
ITO	1.615	4.146	-0.810	0.0029	0.344	5.566	-0.141	0.0031	0.038	1.665	0.011	0.00012
Phosphorene	0.909	3.277	-0.456	0.0036	0.354	6.284	-0.146	0.0030	0.032	1.108	0.008	0.00007
Silicene	0.820	3.033	-0.412	0.0041	0.362	6.718	-0.151	0.0028	0.030	0.988	0.007	0.000064
MoS_2	0.888	3.200	-0.446	0.0038	0.362	6.698	-0.151	0.0027	0.031	1.086	0.008	0.000069
hBN	1.253	3.741	-0.628	0.0035	0.355	6.304	-0.146	0.0027	0.037	1.565	0.010	0.0001
Graphene	1.425	3.775	-0.716	0.0029	0.347	5.701	-0.143	0.0028	0.046	2.698	0.014	0.00022
Black phosphorus	0.890	3.148	-0.446	0.0041	0.381	7.511	-0.160	0.0029	0.033	1.253	0.009	0.000076
AlN	1.796	4.157	-0.901	0.0030	0.357	6.318	-0.148	0.0028	0.044	2.458	0.013	0.00019

Table S6: The fitting constants A_1, A_2, and A_3 describe the normalized magnetization dynamics in 90 nm thick CGT, Cr_3, and FGT samples. A_1 corresponds to the magnetization after the system reaches temporal equilibrium between the thermal baths, while A_2 captures the initial rise in electron temperature. A_3 accounts for state filling amplitude. Root Mean Square Error (RMSE) measures the discrepancy between the simulated data and the fitted model.

Thickness	Timescale	CGT			CrI_3			FGT		
		α (ns $[\kappa_p]^{-n}$)	n	τ^0 (ns)	α (ns $[\kappa_p]^{-n}$)	n	τ^0 (ns)	α (ns $[\kappa_p]^{-n}$)	n	τ^0 (ns)
14 nm	τ_e	-0.00178	0.361	0.0049	0.000041	1.0	0.0041	0.0000065	1.01	0.00016
	τ_m	-0.108	0.273	0.266	-0.00985	0.686	0.0853	0.0210	0.150	0.00253
	$\tau_{m,re}$	-3.7	0.275	8.46	-4329.52	0.000131	4332.11	-107.55	0.00255	108.19
90 nm	τ_e	-2.89	0.000191	2.93	0.000281	0.251	0.00407	-0.0000139	0.646	0.000132
	τ_m	-0.00282	1.0	0.564	0.00231	0.823	0.11	-0.00011	1.0	0.00244
	$\tau_{m,re}$	-590.95	0.000207	592.127	-777.39	0.274	778.853	-1.55	0.142	2.42

Table S7: We used a power law equation ($\alpha\kappa_p^n + \tau^0$) to fit the scattered data for the timescales (τ_e, τ_m, and $\tau_{m,re}$) on 14 nm and 90 nm thickness of CGT, CrI_3, and FGT for Figure 3 in the main text. In the table, $[\kappa_p] = $ W m^{-1}K^{-1} represents the units which is used for simplification.

Extracted timescales by fitting with 3TM model

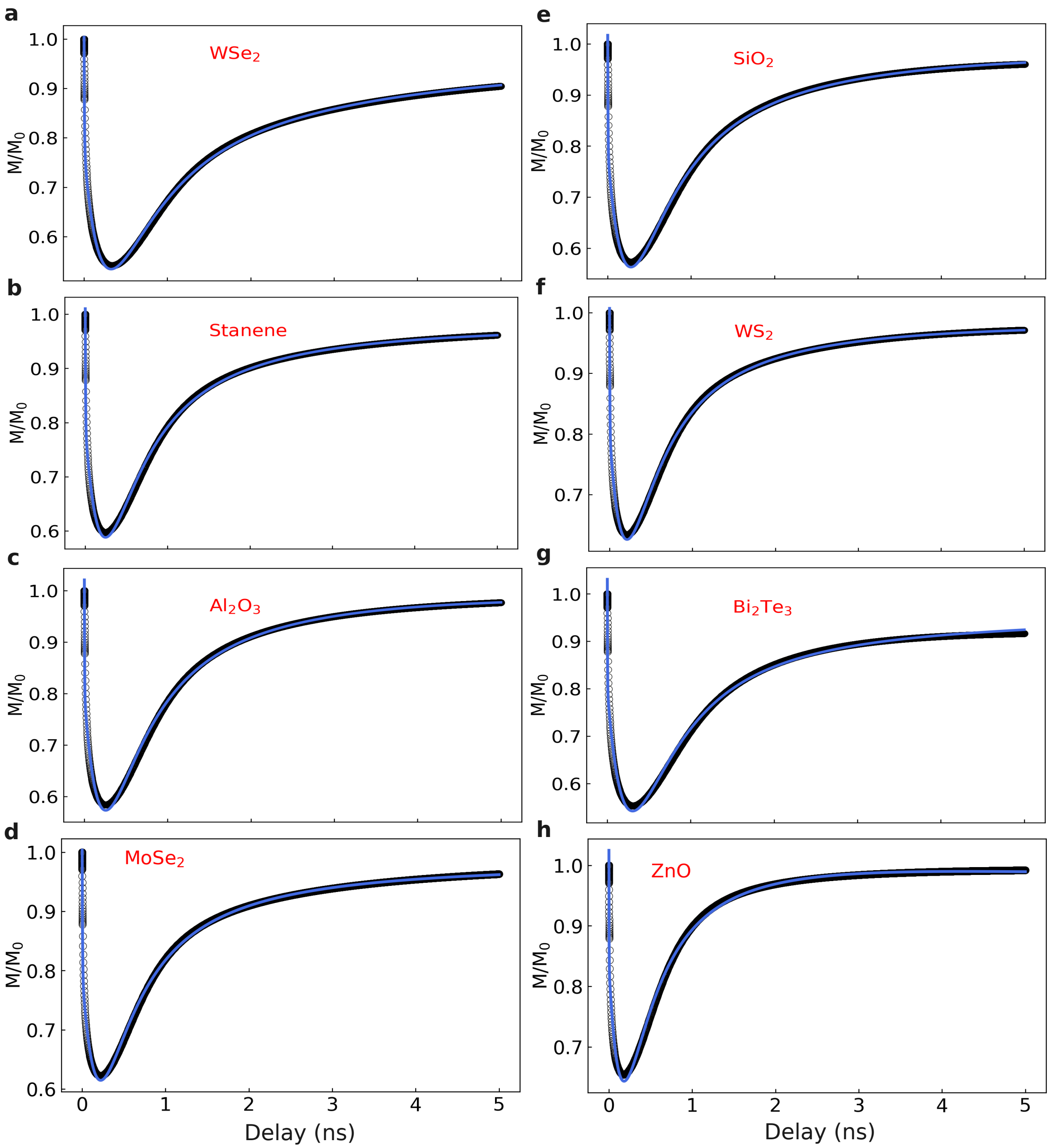

Figure S16: Normalized magnetization as a function of decay time after laser excitation with a fluence of 0.3 mJ/cm^2 at 6 K for a 14 nm thick CGT sample on various substrates: (a) WSe_2, (b) Stanene, (c) Al_2O_3, (d) $MoSe_2$, (e) SiO_2, (f) WS_2, (g) Bi_2Te_3, and (h) ZnO. Timescales are extracted by fitting (blue symbols) with the analytical solution of the 3TM model.

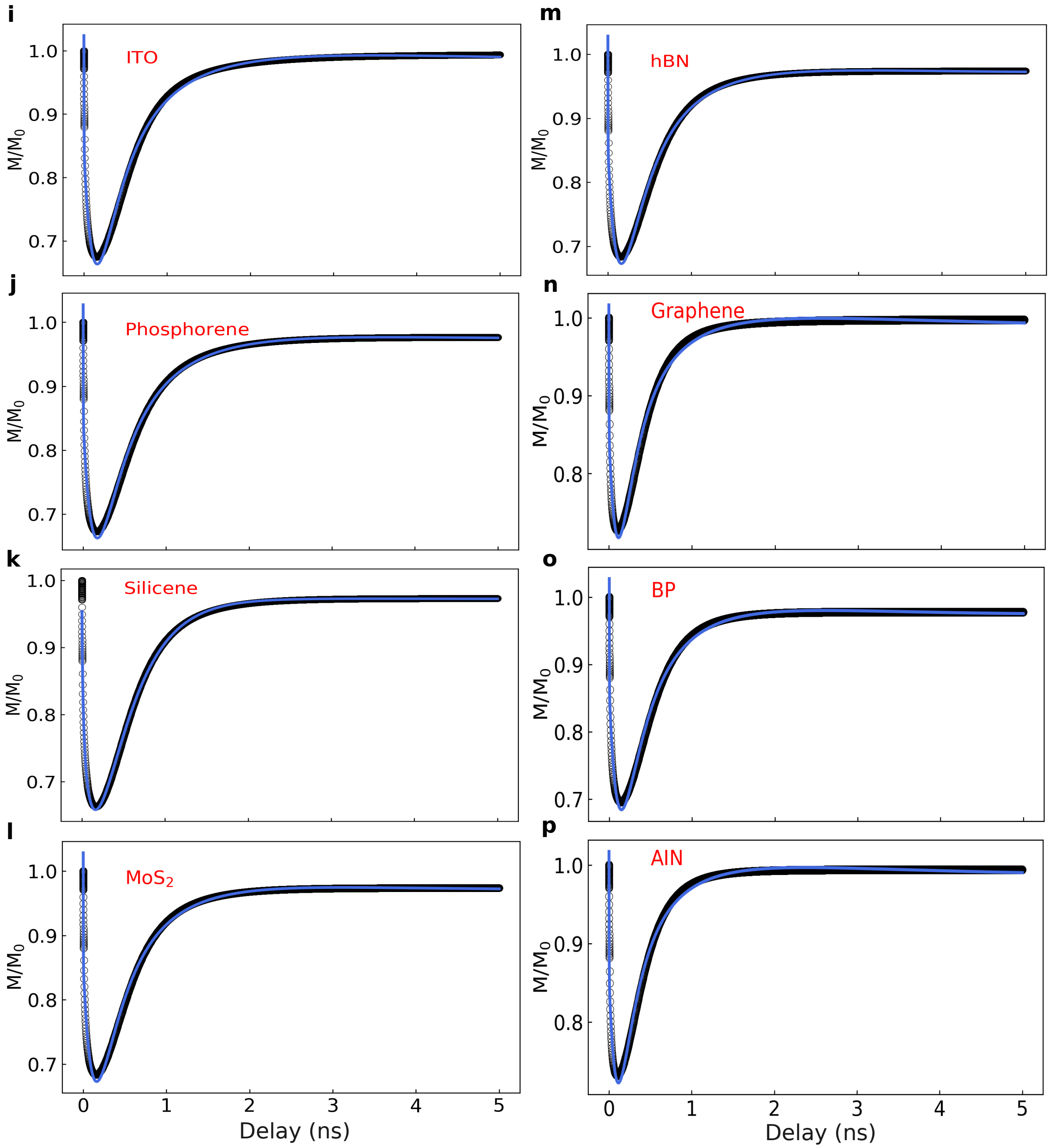

Figure S17: Normalized magnetization as a function of decay time after laser excitation with a fluence of 0.3 mJ/cm^2 at 6 K for a 14 nm thick CGT sample on various substrates: (i) ITO, (j) Phosphorence, (k) Silicene, (l) MoS_2, (m) hBN, (n) Graphene, (o) Black Phosphorus, and (p) AIN. Timescales are extracted by fitting (blue symbols) with the analytical solution of the 3TM model.

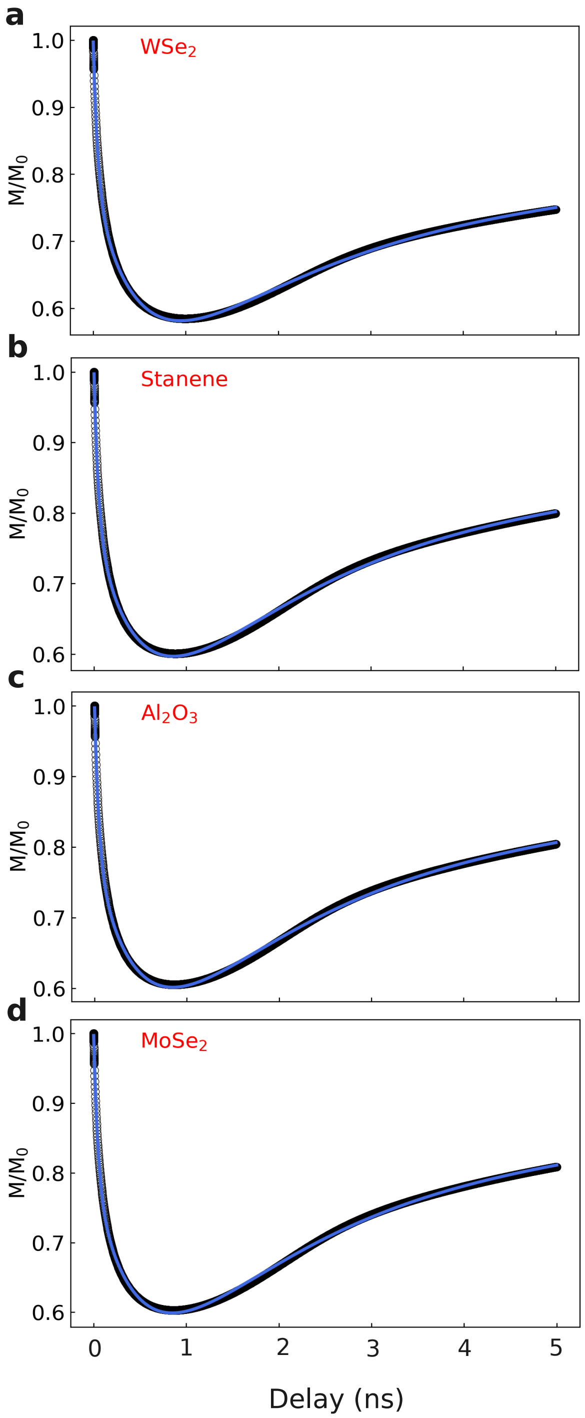

Figure S18: Normalized magnetization as a function of decay time after laser excitation with a fluence of 0.3 mJ/cm^2 at 6 K for a 90 nm thick CGT sample on various substrates: (a) WSe_2, (b) Stanene, (c) Al_2O_3, (d) $MoSe_2$, (e) SiO_2, (f) WS_2, (g) Bi_2Te_3, and (h) ZnO. Timescales are extracted by fitting (blue symbols) with the analytical solution of the 3TM model.

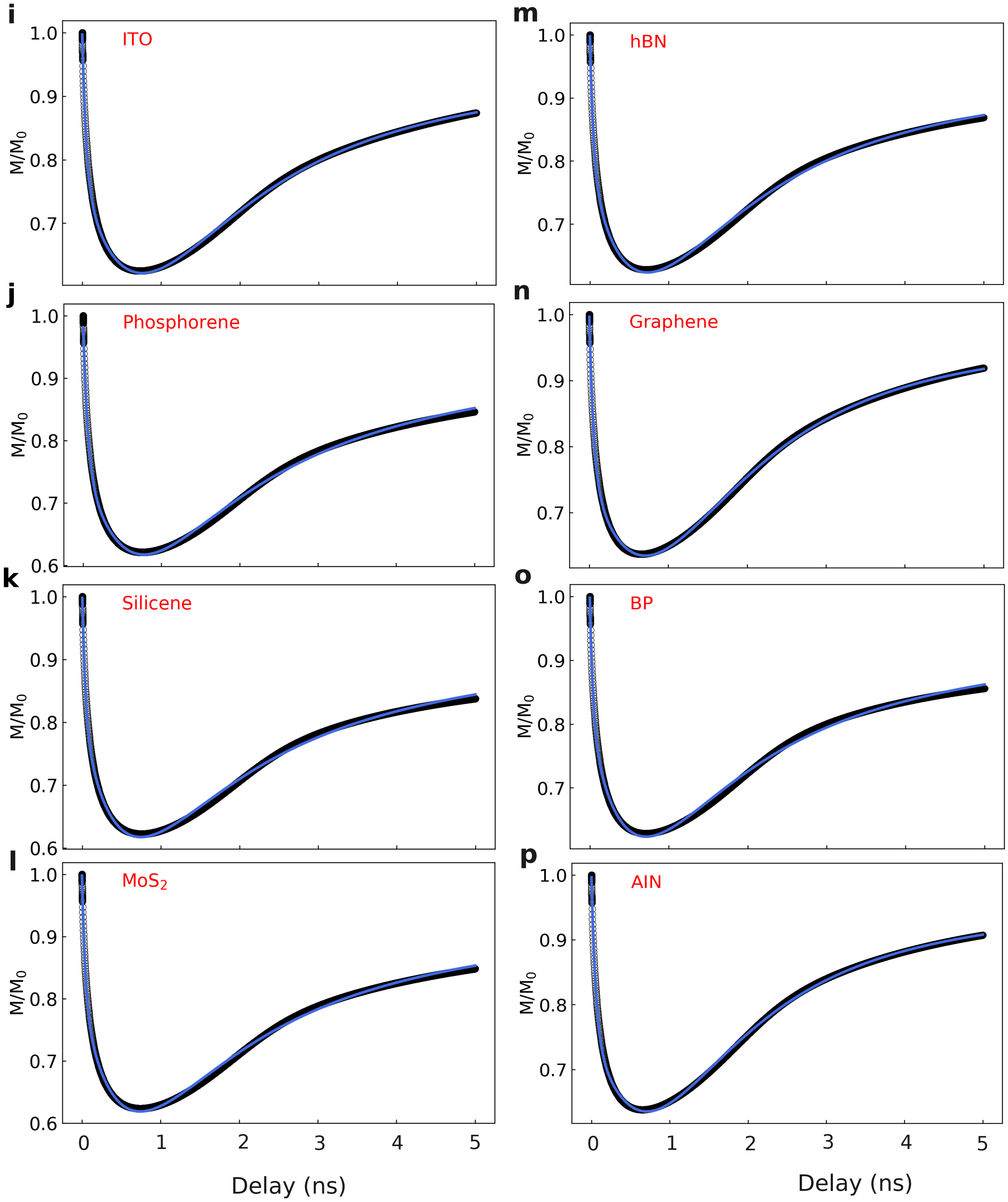

Figure S19: Normalized magnetization as a function of decay time after laser excitation with a fluence of 0.3 mJ/cm^2 at 6 K for a 90 nm thick CGT sample on various substrates: (i) ITO, (j) Phosphorence, (k) Silicene, (l) MoS_2, (m) hBN, (n) Graphene, (o) Black Phosphorus, and (p) AlN. Timescales are extracted by fitting (blue symbols) with the analytical solution of the 3TM model.

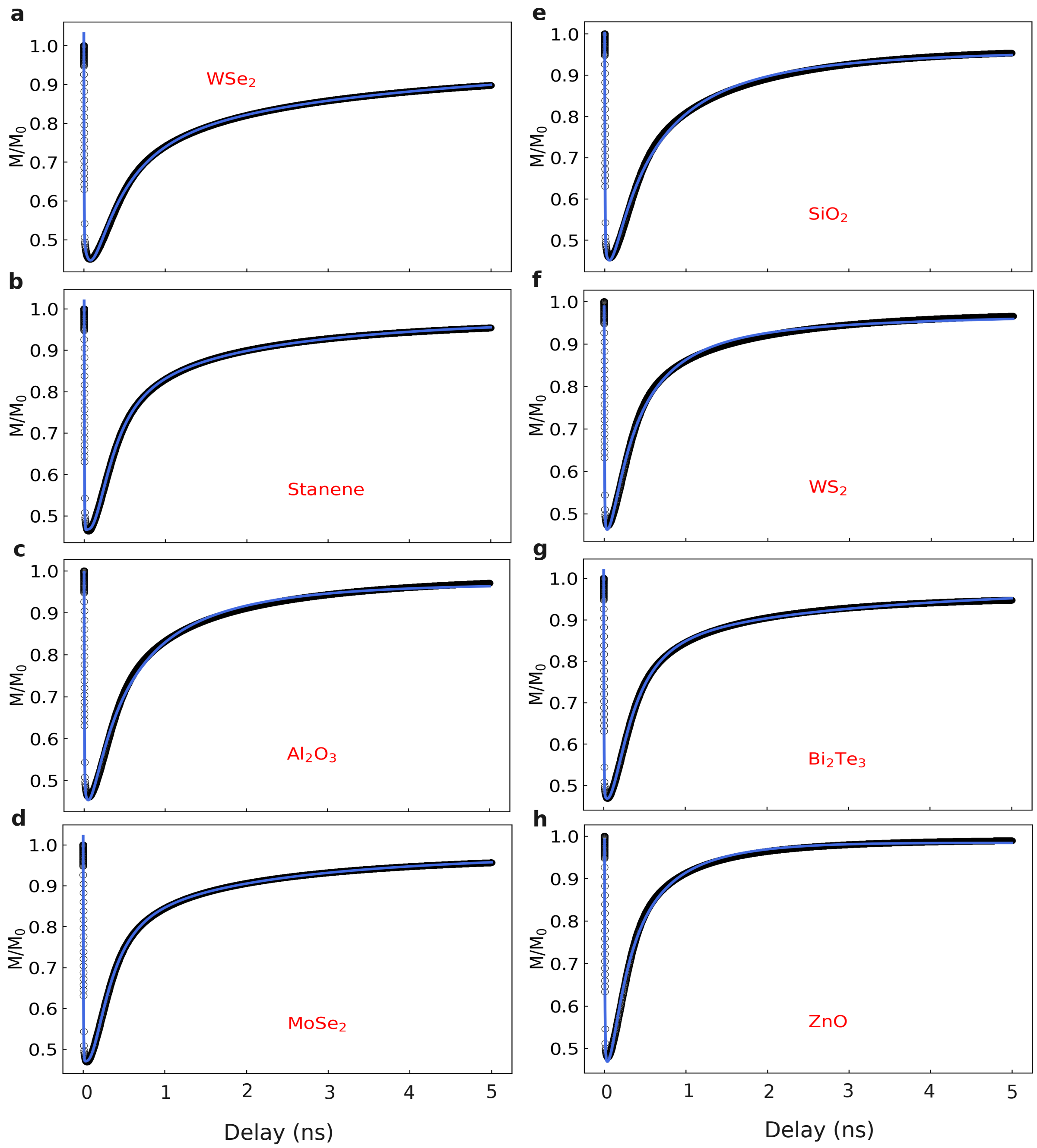

Figure S20: Normalized magnetization as a function of decay time after laser excitation with a fluence of 0.3 mJ/cm^2 at 6 K for a 14 nm thick CrI_3 sample on various substrates: (a) WSe_2, (b) Stanene, (c) Al_2O_3, (d) $MoSe_2$, (e) SiO_2, (f) WS_2, (g) Bi_2Te_3, and (h) ZnO. Timescales are extracted by fitting (blue symbols) with the analytical solution of the 3TM model.

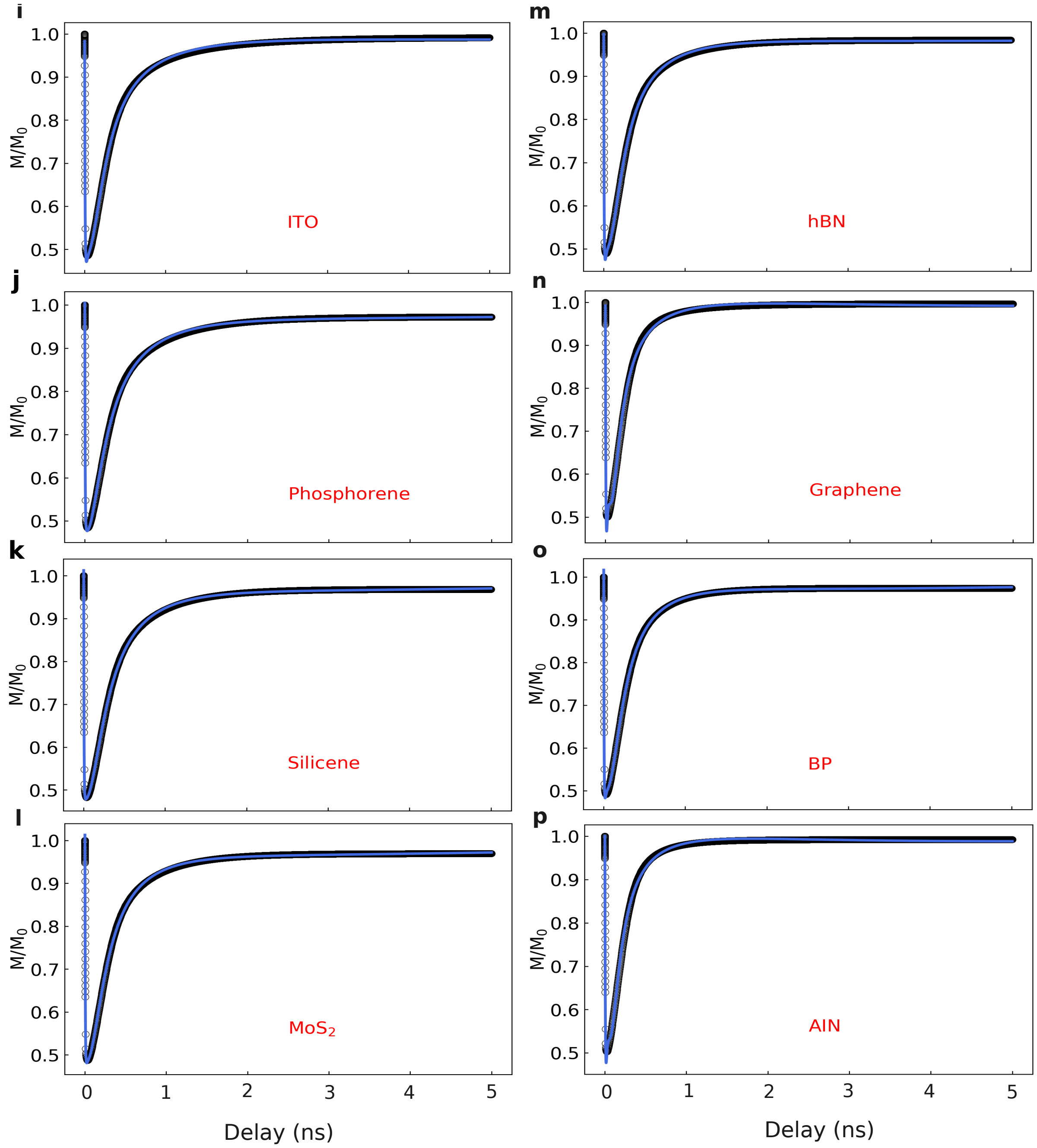

Figure S21: Normalized magnetization as a function of decay time after laser excitation with a fluence of 0.3 mJ/cm^2 at 6 K for a 14 nm thick CrI_3 sample on various substrates: (i) ITO, (j) Phosphorence, (k) Silicene, (l) MoS_2, (m) hBN, (n) Graphene, (o) Black Phosphorus, and (p) AlN. Timescales are extracted by fitting (blue symbols) with the analytical solution of the 3TM model.

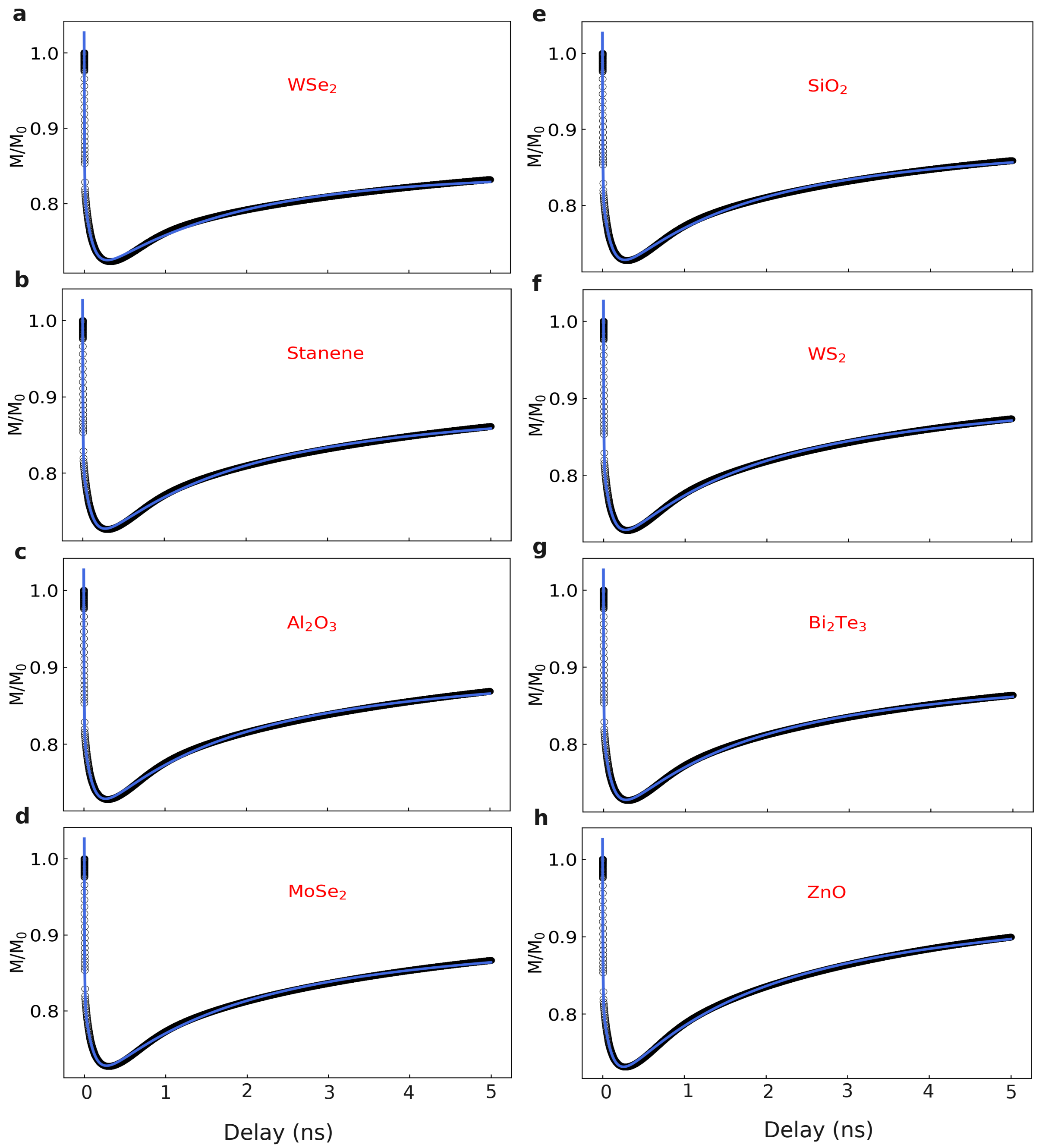

Figure S22: Normalized magnetization as a function of decay time after laser excitation with a fluence of 0.3 mJ/cm^2 at 6 K for a 90 nm thick CrI_3 sample on various substrates: (a) WSe_2, (b) Stanene, (c) Al_2O_3, (d) $MoSe_2$, (e) SiO_2, (f) WS_2, (g) Bi_2Te_3, and (h) ZnO. Timescales are extracted by fitting (blue symbols) with the analytical solution of the 3TM model.

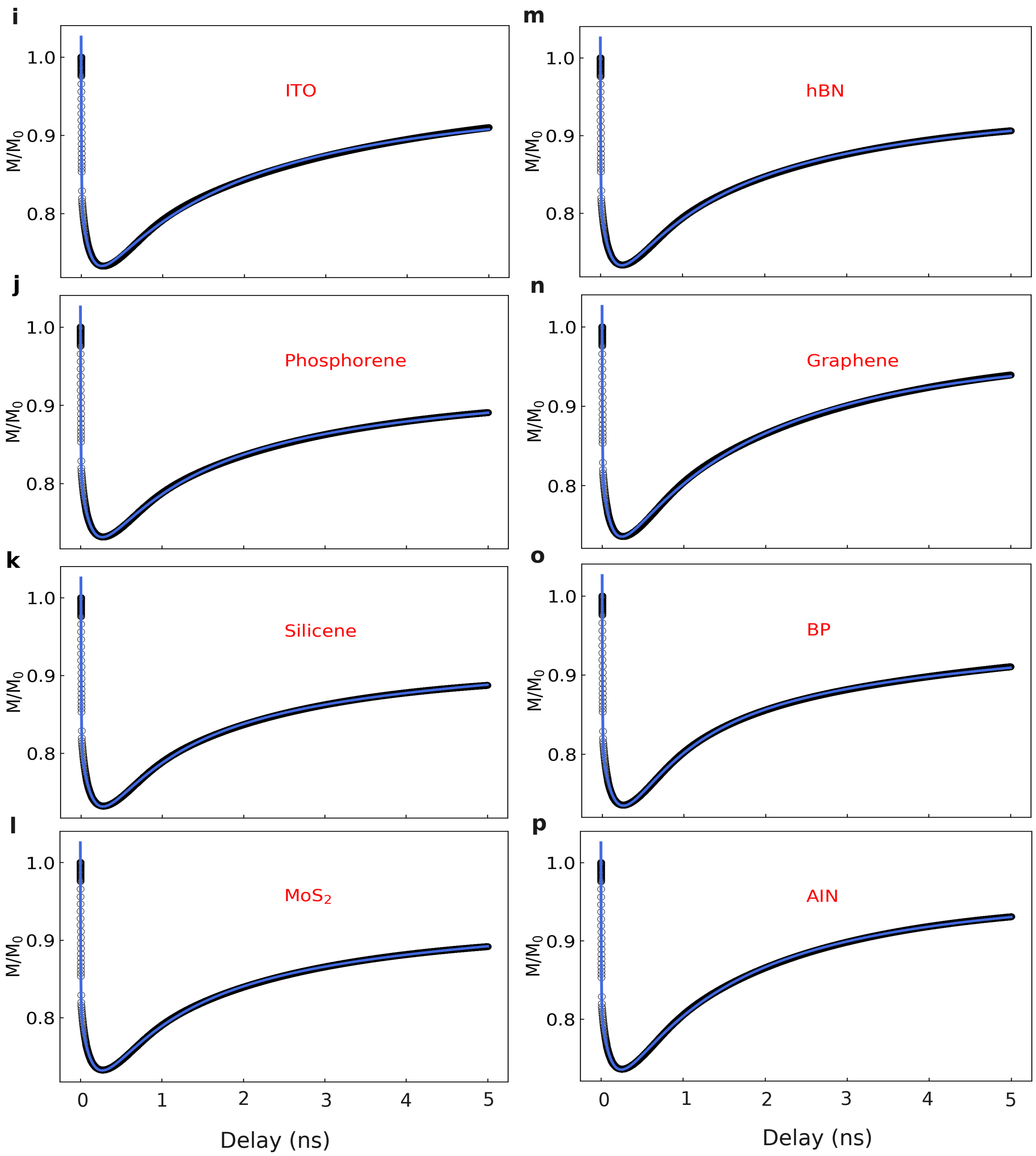

Figure S23: Normalized magnetization as a function of decay time after laser excitation with a fluence of 0.3 mJ/cm^2 at 6 K for a 90 nm thick CrI_3 sample on various substrates: (i) ITO, (j) Phosphorence, (k) Silicene, (l) MoS_2, (m) hBN, (n) Graphene, (o) Black Phosphorus, and (p) AIN. Timescales are extracted by fitting (blue symbols) with the analytical solution of the 3TM model.

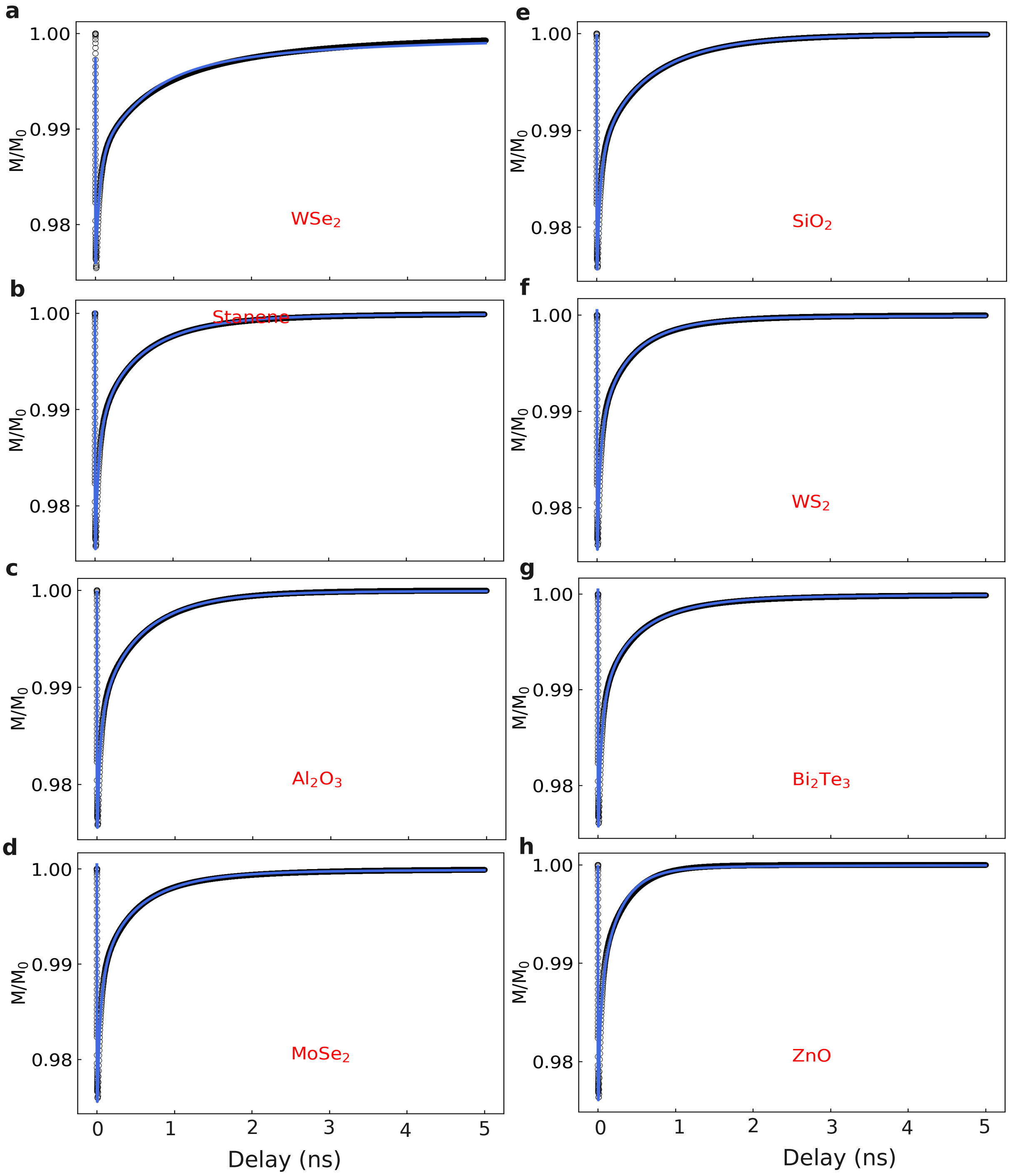

Figure S24: Normalized magnetization as a function of decay time after laser excitation with a fluence of 0.3 mJ/cm^2 at 6 K for a 14 nm thick FGT sample on various substrates: (a) WSe_2, (b) Stanene, (c) Al_2O_3, (d) $MoSe_2$, (e) SiO_2, (f) WS_2, (g) Bi_2Te_3, and (h) ZnO. Timescales are extracted by fitting (blue symbols) with the analytical solution of the 3TM model.

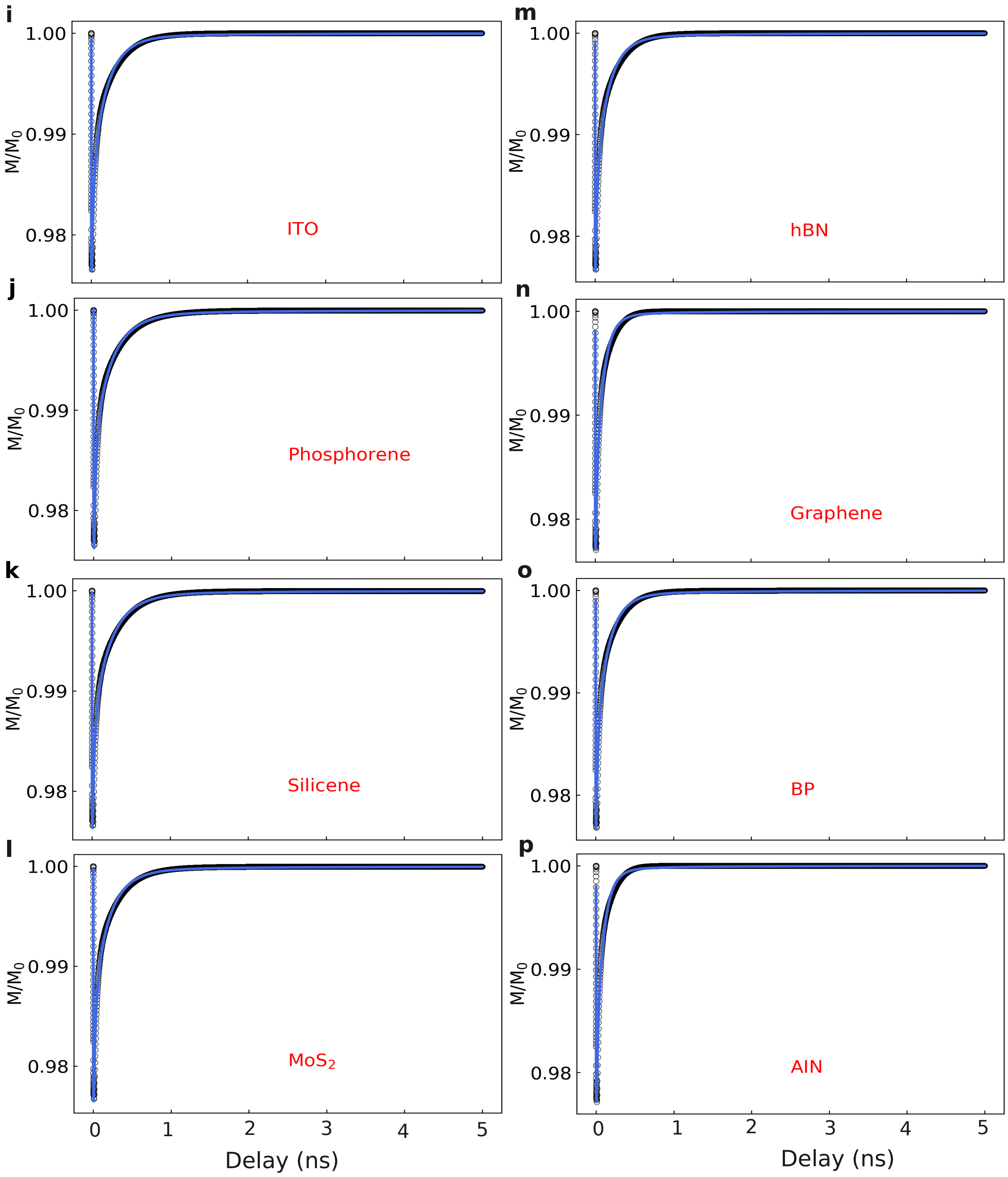

Figure S25: Normalized magnetization as a function of decay time after laser excitation with a fluence of 0.3 mJ/cm^2 at 6 K for a 14 nm thick FGT sample on various substrates: (i) ITO, (j) Phosphorence, (k) Silicene, (l) MoS_2, (m) hBN, (n) Graphene, (o) Black Phosphorus, and (p) AIN. Timescales are extracted by fitting (blue symbols) with the analytical solution of the 3TM model.

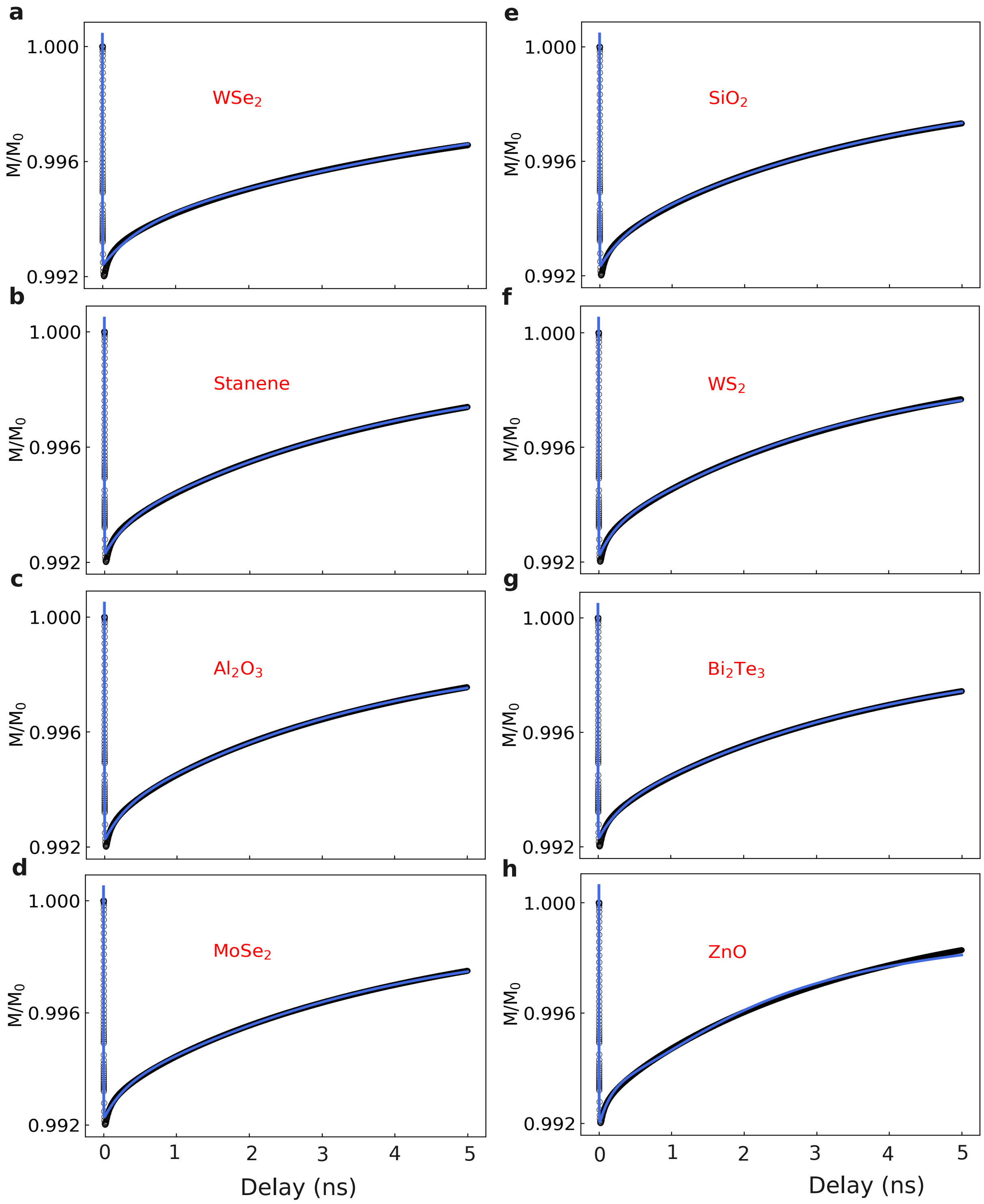

Figure S26: Normalized magnetization as a function of decay time after laser excitation with a fluence of 0.3 mJ/cm^2 at 6 K for a 90 nm thick FGT sample on various substrates: (a) WSe_2, (b) Stanene, (c) Al_2O_3, (d) $MoSe_2$, (e) SiO_2, (f) WS_2, (g) Bi_2Te_3, and (h) ZnO. Timescales are extracted by fitting (blue symbols) with the analytical solution of the 3TM model.

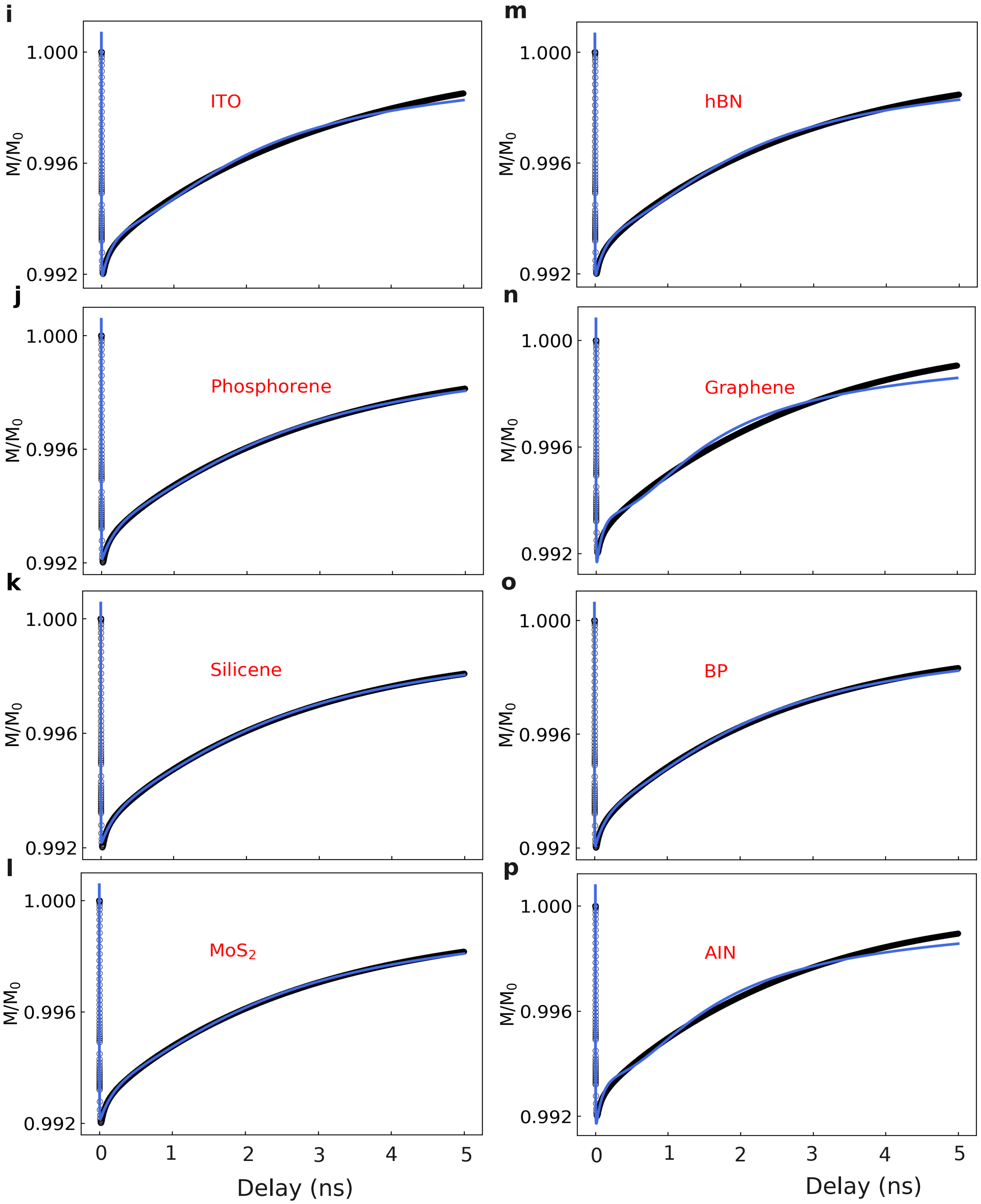

Figure S27: Normalized magnetization as a function of decay time after laser excitation with a fluence of 0.3 mJ/cm^2 at 6 K for a 90 nm thick FGT sample on various substrates: (i) ITO, (j) Phosphorence, (k) Silicene, (l) MoS_2, (m) hBN, (n) Graphene, (o) Black Phosphorus, and (p) AIN. Timescales are extracted by fitting (blue symbols) with the analytical solution of the 3TM model.

7 Non-thermal nature of spin dynamics

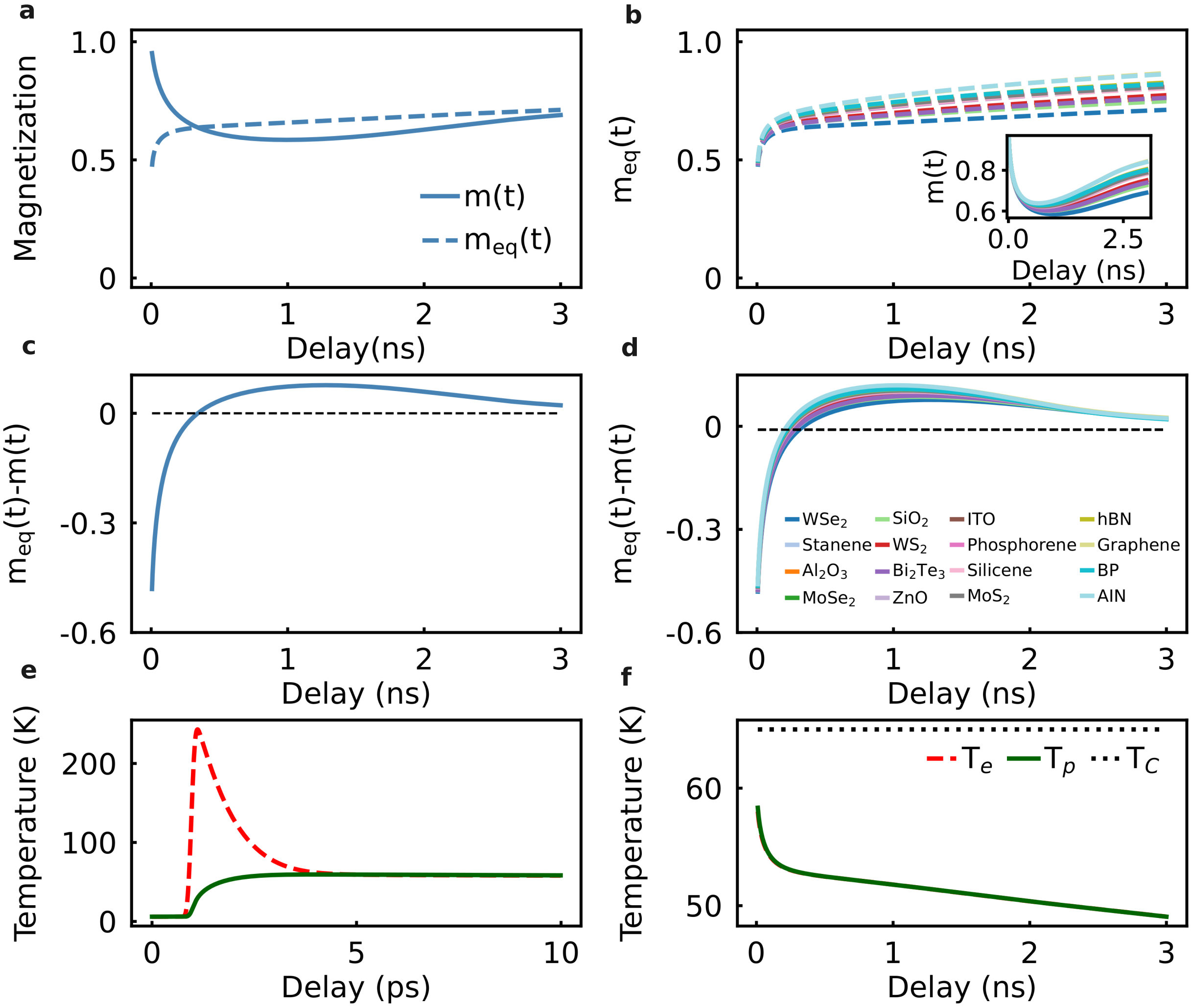

Figure S28: Illustration of non-Thermal Behavior in 90 nm thick CGT. **a** shows the average magnetization dynamics $m(t)$ (solid line) and equilibrium magnetization $m_{\text{eq}}(t)$ (dashed line) for the heterostructure with hBN/CGT/WSe_2. **b** displays the average magnetization dynamics $m(t)$ for various substrates considered in our simulations. The inset of **b** shows the equilibrium magnetization $m_{\text{eq}}(t)$ for all the substrates. In the middle panels, the difference between $m_{\text{eq}}(t)$ and $m(t)$ is presented in **c** for the substrate WSe_2 and **d** for all the substrates. The bottom panels **e** and **f** depict the evolution of electron and phonon temperatures, with the dotted line marking the Curie temperature of CGT.

8 On the normalization on dm/dt

We note that the signal dm/dt in Figures 4.e-f) of the main text have been normalized to the maximum value of $|dm/dt|$ of all simulations shown within the same panel, yielding a unitless signal. Maximum demagnetization occurs right after pulse excitation and the remagnetization signal is significantly weaker, thus we see the small values achieved in the shown figures of the remagnetization process.

9 Connection between magnetization dynamics and electromagnetic field stimulation

As outlined in previous works [53,54], the ultrafast magnetization rate dm/dt can be interpreted as an exchange of angular momentum between localized spins with magnetization m and itinerant spins with a chemical potential shift between up and down spin states, the spin accumulation μ_s. Their angular momentum exchange is manifested in the relation

$$\frac{d\mu_s}{dt} \propto \frac{dm}{dt} \tag{1}$$

We shall add that the first order Taylor expansion of the M3TM for spin S=1/2 as stated in Eq.(3) in the main text is derived the following way:

$$\begin{aligned}\mathscr{T}_1\left(\frac{dm}{dt}\right) &= R\frac{T_p}{T_C}(B_S - \frac{B_S^2}{B_S}) \\ &\quad + R\frac{T_p}{T_C}(1 - 2\frac{B_S}{B_S} - B_S')(m - B_S) \\ &= -R\frac{T_p}{T_C}(1 + B_S')(m - B_S)\end{aligned}$$

The spin accumulation of itinerant electrons has two dissipation channels, e.g. spin flip relaxation (M3TM) and spin current propagation. We define here the spin current in the diffusive regime as done in previous works on the s-d-exchange model [55]:

$$\frac{d\mu_s}{dt}|_{\text{diss}} = \frac{d\mu_s}{dt}|_{sf} + D\Delta\mu_s, \tag{2}$$

where D is the spin diffusion constant and Δ is the Laplace operator. In magnetic thin films on insulating substrates the diffusive term is usually negligible since the spin polarized current cannot diffuse out of the magnetic sample. Under the assumption of strong coupling of m and μ_s, the magnetization dynamics are driven by the spin flip rate described with the M3TM. In contact with conductive substrates however, spin accumulation that arises close to the interface can diffuse into the substrate, resulting in a second, non-local channel of angular momentum dissipation and a spin current $j_s(t)$ in the substrate. Via the inverse spin-Hall effect, the spin current absorbed by the conducting substrate can be converted into a perpendicular charge current $j_c(t) \propto j_s(t)$ in the presence of spin-orbit coupling [53]. Just as the spin current, the charge current switches sign upon the transition between demagnetization and remagnetization phases and thus emit a time-dependent electromagnetic pulse $E_M(t)$, for which holds:

$$E_M(t) \propto j_c(t) \propto j_s(t) \propto \frac{d\mu_s}{dt} \propto \frac{dm}{dt} \tag{3}$$

Alltogether, the magnetization dynamics directly stimulate the excitation frequency of the electro-magnetic pulse. The Fourier transform of the magnetization rate dm/dt then yields the frequency spectrum of the electromagnetic pulse, driven by the spin accumulation that arises from the magnetization dynamics:

$$\mathscr{F}(E_M)(\omega) \propto \mathscr{F}(\frac{dm}{dt})(\omega) \tag{4}$$

In Type I magnetization dynamics, the dominant frequency of the electromagnetic pulse lies in the Terahertz (THz) regime, because of rapid remagnetization after a few picoseconds (ps). The dominant frequency here denotes the frequency at which the Fourier transforms finds its maximum. The 2D materials studied in this manuscript show Type II magnetization dynamics, where the remagnetization is driven by thermal diffusion into adjacent substrates on nanosecond (ns) timescales. Thus, depending on thickness and choice of the ferromagnet, as well as the choice of substrate, the Fourier transform of dm/dt has dominant frequencies of hundreds of Megahertz (MHz) to a few Gigahertz (GHz). The dominant frequencies depicted in Figure (**??**) were calculated by finding the maximum of

$$\mathscr{F}(\frac{dm}{dt})(\omega) = \int_0^{t_{max}} dt\ e^{i\omega t}, \tag{5}$$

where $\omega = 2\pi f$ and f is the frequency. t_{max} is determined by the time at which the magnetization dynamics have equilibrated and $dm/dt = \delta m = 0$ for all later times.

10 Light Absorption and Heat Profile Differences

Figure S29 presents the effect of laser penetration depth on the temperature profiles of Fe_3GeTe_2 (FGT) and $Cr_2Ge_2Te_6$ (CGT) for two different sample thicknesses 14 nm and 90 nm. The results demonstrate distinct thermal behaviors in metallic (FGT) and semiconducting (CGT) vdW magnets due to their different absorption and heat dissipation mechanisms.

In thin samples (14 nm), where the sample thickness is smaller than the laser penetration depth, a significant fraction of the laser energy is transmitted through the material, leading to lower overall absorbed energy. This results in a more pronounced demagnetization in CGT due to its larger effective heating region, whereas in FGT, the demagnetization remains surface-confined due to its metallic nature and rapid electron-lattice equilibration.

For thicker samples (90 nm), the penetration depth plays a crucial role in heat distribution. For penetration depths of 14 nm and 30 nm, demagnetization is weaker as the absorbed energy is distributed over a larger volume, reducing the peak temperature increase. However, for larger penetration depths (60 nm and 80 nm), demagnetization becomes more pronounced, as energy is absorbed more effectively within the magnetic volume. This suggests that in insulating vdW magnets, there exists an optimal penetration depth that maximizes thermal effects on magnetization dynamics by balancing heat absorption and distribution. In contrast, FGT does not exhibit this trend, as its metallic nature ensures that heat remains confined within the first few nanometers, leading to similar demagnetization behavior in both thicknesses.

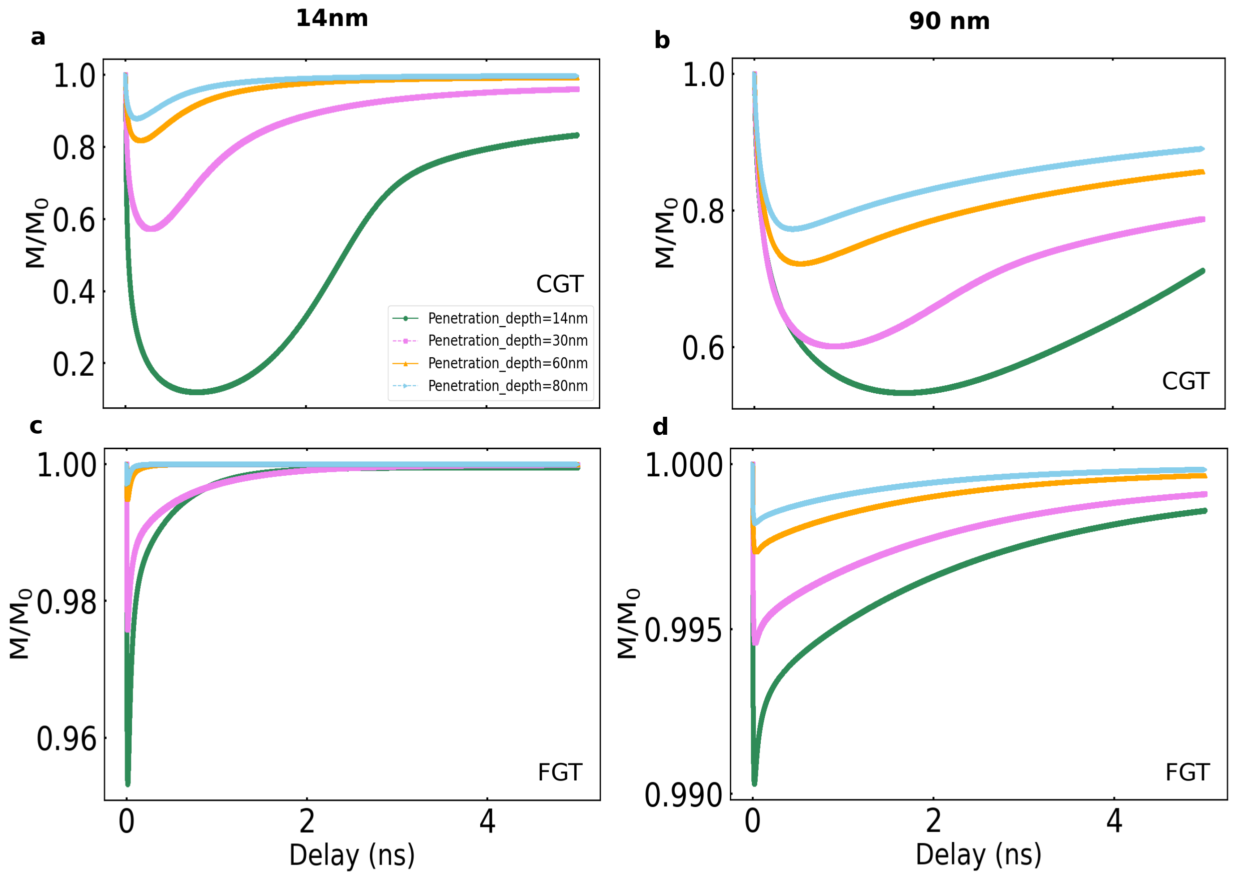

Figure S29: Effect of laser penetration depth on demagnetization in Fe_3GeTe_2 (FGT) and $Cr_2Ge_2Te_6$ (CGT) for two different sample thicknesses (14 nm and 90 nm)

Note that in 90 nm samples, the full pulse energy is absorbed for all shown penetration depths. Here, smaller penetration depths yield an accumulation of thermal energy towards the sample-surface and the heat takes longer to leave the magnetic material through the interface to the substrate, yielding a more pronounced demagnetization.

11 Green's function analysis of the timescales of in-plane and out-of-plane heat diffusion

Here, we investigate briefly the ratio of in-plane and out-of-plane heat flow in quasi-two-dimensional van der Waals materials upon excitation with an ultrafast laser pulse, characterized by it's spot-size w_p and penetration depth λ. The temperature dynamics in the diffusive regime are governed by the continuity equation for the temperature T as

$$C\,\partial_t T = \nabla(\kappa \nabla T), \tag{6}$$

where C denotes the heat capacity, which we assume constant for the purpose of this analysis. To separate the in-plane (ip) and out-of-plane (oop) contributions to the heat flow, the temperature profiles due to laser absorption are estimated as a gaussian ip distribution along the x direction and an exponentially decaying oop distribution along the z direction:

$$T(x,z) = T_0 \exp(-\frac{x^2}{2w_p^2}) \exp(-\frac{z}{\lambda}) + T_i, \tag{7}$$

where T_i denotes the initial temperature before pulse excitation. From this relation one can readily find the ratio $q = \frac{\partial_t T|_{ip}}{\partial_t T|_{oop}}$ of temperature changes due to ip and oop heat diffusion using Eq. (6):

$$q(x,z) = \frac{\frac{\kappa_{ip}}{w_p^4}(x^2 - w_p^2)\exp(-\frac{x^2}{2w_p^2})}{\frac{\kappa_{oop}}{\lambda^2}\exp(-\frac{z}{\lambda})} \tag{8}$$

We are interested in the temperature dynamics close to the center of the pulse excitation, where the magnetization dynamics are measured, for example with MOKE technique. For $x = z = 0$ we find

$$|q(0,0)| = \frac{\kappa_{ip}\lambda^2}{\kappa_{oop}w_p^2} \tag{9}$$

The long-time behaviour of the gaussian *ip* and exponential *oop* temperature distributions can be approximated by a Green's function analysis of Eq. (6). The Green's function solving Eq. (6) for the initial condition $G(\mathbf{x} = 0) = \delta(\mathbf{x})$ can be analytically found by realizing that for the Fourier transform $\tilde{G}(k)$ holds

$$\partial_t \tilde{G}(k) = -k^2 \tilde{G}(k) \tag{10}$$

Back transformation yields an ordinary differential equation and one finds the gaussian

$$G(x,t) = \frac{1}{\sqrt{4\pi\frac{\kappa}{C}t}} \exp\left(-\frac{x^2}{4\frac{\kappa}{C}t}\right). \tag{11}$$

We set initial conditions for the in-plane (ip) and out-of plane (oop) temperature profiles following the laser absorption profile P, since this term dominates the temperature dynamics for small time delays. The temperature distributions over time can be found by

$$T(x,t) = \int dx\, G(x-x',t)\, T(x',0) \tag{12}$$

The ip distribution is computed by folding gaussians, which yields a gaussian of the added variances:

$$T_{ip}(t) \propto \exp\left(-\frac{x^2}{2(2\frac{\kappa_{ip}}{C}t+w_p^2)}\right), \tag{13}$$

with an effective timescale $\tau_{ip} = w_p^2/2\frac{\kappa}{C}$. While convolution of Green's function with an exponential decay does not yield an analytical result, we realize that the temperature profile follows the pulse excitation and thus defines the effective diffusion length

$$\lambda = \sqrt{\frac{\kappa_{oop}}{C}t} \tag{14}$$

Comparing the timescales, we find

$$\frac{\tau_{ip}}{\tau_{oop}} = \frac{w_p^2 \kappa_{oop}}{2\lambda^2 \kappa_{ip}} \tag{15}$$

Both estimations show, that the *oop* heat flow dominates the diffusion dynamics for pulse widths $w_p \approx 2 - 50\ \mu$m and penetration depths in the range $\lambda \approx 10 - 30$ nm despite the fact that the thermal conductivity is generally much larger in plane $\kappa_{ip} \approx 10\ \kappa_{oop}$.

12 Comparison of model parameters to experimental ultrafast magnetization dynamics

To support the choice of model parameters used in this study, we show here fits of our model to ultrafast magnetization dynamics measured in various experiments. Here, we simulate the magnetic materials as monolayers to recover the parameters defining electron-phonon-interaction and spin-flip rate. These parameters were then chosen to extend the study to longer timescales and investigate diffusion effects.

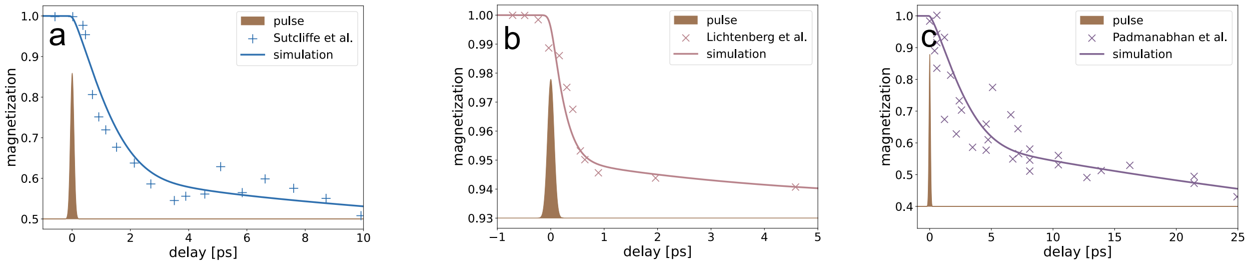

Figure S30: Fits to measured magnetization dynamics in three ferromagnetic van der Waals materials. a) CGT with measurements form Sutcliffe et al. [1]. b) FGT with measurements from Lichtenberg et al. [3]. c) CrI3 with measurements form Padmanabhan et al. [2]. The pump pulse is shown with arbitrary pump intensity to visualize the pulse width and zero-delay.

13 Supplementary References

1. Sutcliffe, E. *et al.* Transient magneto-optical spectrum of photoexcited electrons in the van der Waals ferromagnet $Cr_2Ge_2Te_6$. *Phys. Rev. B* **107**, 174432 (2023). URL `https://link.aps.org/doi/10.1103/PhysRevB.107.174432`.

2. Padmanabhan, P. *et al.* Coherent helicity-dependent spin-phonon oscillations in the ferromagnetic van der Waals crystal CrI_3. *Nature Communications* **13**, 4473 (2022). URL `https://doi.org/10.1038/s41467-022-31786-3`.

3. Lichtenberg, T. *et al.* Anisotropic laser-pulse-induced magnetization dynamics in van der Waals magnet Fe_3GeTe_2. *2D Materials* **10**, 015008 (2022). URL `https://dx.doi.org/10.1088/2053-1583/ac9dab`.

4. Spachmann, S. *et al.* Strong effects of uniaxial pressure and short-range correlations in $Cr_2Ge_2Te_6$. *Physical Review Research* **4**, L022040 (2022).

5. McGuire, M. A., Dixit, H., Cooper, V. R. & Sales, B. C. Coupling of crystal structure and magnetism in the layered, ferromagnetic insulator CrI_3. *Chemistry of Materials* **27**, 612–620 (2015).

6. Hwang, I. *et al.* Hard ferromagnetic van-der-Waals metal (Fe, Co)$_3$GeTe$_2$: a new platform for the study of low-dimensional magnetic quantum criticality. *Journal of Physics: Condensed Matter* **31**, 50LT01 (2019).

7. Zhu, J.-X. *et al.* Electronic correlation and magnetism in the ferromagnetic metal Fe_3GeTe_2. *Physical Review B* **93**, 144404 (2016).

8. Claro, M. S. *et al.* Temperature and Thickness Dependence of the Thermal Conductivity in 2D Ferromagnet Fe_3GeTe_2. *ACS applied materials & interfaces* **15**, 49538–49544 (2023).

9. Hatayama, S., Yagi, T. & Sutou, Y. Mixed-conduction mechanism of $Cr_2Ge_2Te_6$ film enabling positive temperature dependence of electrical conductivity and seebeck coefficient. *Results in Materials* **8**, 100155 (2020).

10. Bolgar, A., Trofimova, Z. A. & Yanaki, A. Thermodynamic properties of tungsten diselenide in a broad temperature range. *Soviet Powder Metallurgy and Metal Ceramics* **29**, 382–385 (1990).

11. Mathew, S. *et al.* Temperature dependent structural evolution of wse2: A synchrotron x-ray diffraction study. *Condensed Matter* **5**, 76 (2020).

12. Norouzzadeh, P. & Singh, D. J. Cross-plane thermal conductivity of tungsten diselenide. *physica status solidi c* **14**, 1700078 (2017).

13. Kuang, Y. D., Lindsay, L., Shi, S. Q. & Zheng, G. Tensile strains give rise to strong size effects for thermal conductivities of silicene, germanene and stanene. *Nanoscale* **8**, 3760–3767 (2016).

14. Nissimagoudar, A. S., Manjanath, A. & Singh, A. K. Diffusive nature of thermal transport in stanene. *Physical Chemistry Chemical Physics* **18**, 14257–14263 (2016).

15. Hong, Y., Han, D., Hou, B., Wang, X. & Zhang, J. High-throughput computations of cross-plane thermal conductivity in multilayer stanene. *International Journal of Heat and Mass Transfer* **171**, 121073 (2021).

16. Calvin, J. J., Asplund, M., Zhang, Y., Huang, B. & Woodfield, B. F. Heat capacity and thermodynamic functions of γ-Al_2O_3. *The Journal of Chemical Thermodynamics* **112**, 77–85 (2017).

17. Schauer, A. Thermal expansion, grueneisen parameter, and temperature dependence of lattice vibration frequencies of aluminum oxide. *Canadian Journal of Physics* **43**, 523–531 (1965).

18. Behkam, B., Yang, Y. & Asheghi, M. Thermal property measurement of thin aluminum oxide layers for giant magnetoresistive (gmr) head applications. *International journal of heat and mass transfer* **48**, 2023–2031 (2005).

19. Peng, B. *et al.* Thermal conductivity of monolayer mos_2, $mose_2$, and ws_2: interplay of mass effect, interatomic bonding and anharmonicity. *RSC advances* **6**, 5767–5773 (2016).

20. Rahman, M., Shahzadeh, M. & Pisana, S. Simultaneous measurement of anisotropic thermal conductivity and thermal boundary conductance of 2-dimensional materials. *Journal of Applied Physics* **126** (2019).

21. Engelmann, S. & Hentschke, R. Specific heat capacity enhancement studied in silica doped potassium nitrate via molecular dynamics simulation. *Scientific reports* **9**, 7606 (2019).

22. Callard, S., Tallarida, G., Borghesi, A. & Zanotti, L. Thermal conductivity of SiO_2 films by scanning thermal microscopy. *Journal of non-crystalline solids* **245**, 203–209 (1999).

23. Zhu, W., Zheng, G., Cao, S. & He, H. Thermal conductivity of amorphous SiO_2 thin film: A molecular dynamics study. *Scientific reports* **8**, 10537 (2018).

24. Pisoni, A. *et al.* Anisotropic transport properties of tungsten disulfide. *Scripta Materialia* **114**, 48–50 (2016).

25. Bessas, D. *et al.* Lattice dynamics in bi_2te_3 and sb_2te_3: Te and sb density of phonon states. *Physical Review B—Condensed Matter and Materials Physics* **86**, 224301 (2012).

26. Jena, A., Lee, S.-C. & Bhattacharjee, S. Tuning the lattice thermal conductivity in bismuth telluride via cr alloying. *Physical Review Applied* **15**, 064023 (2021).

27. Norimasa, O. & Takashiri, M. In-and cross-plane thermoelectric properties of oriented Bi_2Te_3 thin films electrodeposited on an insulating substrate for thermoelectric applications. *Journal of Alloys and Compounds* **899**, 163317 (2022).

28. Barin, I. Thermochemical data of pure substances (1989).

29. Xu, Y., Goto, M., Kato, R., Tanaka, Y. & Kagawa, Y. Thermal conductivity of zno thin film produced by reactive sputtering. *Journal of Applied Physics* **111** (2012).

30. Farid, N. *et al.* Improvement of electrical properties of ito thin films by melt-free ultra-short laser crystallization. *Journal of Physics D: Applied Physics* **54**, 185103 (2021).

31. Olson, D. H. *et al.* Size effects on the cross-plane thermal conductivity of transparent conducting indium tin oxide and fluorine tin oxide thin films. *IEEE Transactions on Components, Packaging and Manufacturing Technology* **9**, 51–57 (2018).

32. Chen, W.-H., Chen, I.-C., Cheng, H.-C. & Yu, C.-F. Influence of structural defect on thermal–mechanical properties of phosphorene sheets. *Journal of Materials Science* **52**, 3225–3232 (2017).

33. Qin, G. & Hu, M. Thermal transport in phosphorene. *Small* **14**, 1702465 (2018).

34. Devi, A., Kumar, A., Singh, A. & Ahulwalia, P. A comparative study on phonon spectrum and thermal properties of graphene, silicene and phosphorene. *AIP Conference Proceedings* **2115** (2019).

35. Yang, K., Cahangirov, S., Cantarero, A., Rubio, A. & D'Agosta, R. Thermoelectric properties of atomically thin silicene and germanene nanostructures. *Physical Review B* **89**, 125403 (2014).

36. Barati, M., Vazifehshenas, T., Salavati-Fard, T. & Farmanbar, M. Phononic thermal conductivity in silicene: the role of vacancy defects and boundary scattering. *Journal of Physics: Condensed Matter* **30**, 155307 (2018).

37. Bano, A., Khare, P. & Gaur, N. Thermal transport properties of bulk and monolayer mos2: an ab-initio approach. *Journal of Physics: Conference Series* **836**, 012052 (2017).

38. Gabourie, A. J., Suryavanshi, S. V., Farimani, A. B. & Pop, E. Reduced thermal conductivity of supported and encased monolayer and bilayer mos2. *2D Materials* **8**, 011001 (2020).

39. Sood, A. *et al.* Quasi-ballistic thermal transport across MoS_2 thin films. *Nano letters* **19**, 2434–2442 (2019).

40. Gorbunov, V., Gavrichev, K., Totrova, G., Bochko, A. & Lazarev, V. Thermodynamic properties of β-bn in region of low temperature. *Zhurnal Fizicheskoj Khimii* **61**, 3357–3360 (1987).

41. Du, X., Li, J., Lin, J. & Jiang, H. Temperature dependence of the energy bandgap of multi-layer hexagonal boron nitride. *Applied Physics Letters* **111** (2017).

42. Yuan, C. *et al.* Modulating the thermal conductivity in hexagonal boron nitride via controlled boron isotope concentration. *Communications physics* **2**, 43 (2019).

43. Jiang, P., Qian, X., Yang, R. & Lindsay, L. Anisotropic thermal transport in bulk hexagonal boron nitride. *Physical review materials* **2**, 064005 (2018).

44. Pop, E., Varshney, V. & Roy, A. K. Thermal properties of graphene: Fundamentals and applications. *MRS bulletin* **37**, 1273–1281 (2012).

45. Xie, Y. *et al.* The defect level and ideal thermal conductivity of graphene uncovered by residual thermal reffusivity at the 0 k limit. *Nanoscale* **7**, 10101–10110 (2015).

46. Stephenson, C., Potter, R., Maple, T. & Morrow, J. The thermodynamic properties of elementary phosphorus the heat capacities of two crystalline modifications of red phosphorus, of α and β white phosphorus, and of black phosphorus from 15 to 300 k. *The Journal of Chemical Thermodynamics* **1**, 59–76 (1969).

47. Jeon, S. G., Shin, H., Jaung, Y. H., Ahn, J. & Song, J. Y. Thickness-dependent and anisotropic thermal conductivity of black phosphorus nanosheets. *Nanoscale* **10**, 5985–5989 (2018).

48. Zhang, Y. *et al.* The electrical, thermal, and thermoelectric properties of black phosphorus. *APL Materials* **8** (2020).

49. de Faoite, D., Browne, D. J., Chang-Díaz, F. R. & Stanton, K. T. A review of the processing, composition, and temperature-dependent mechanical and thermal properties of dielectric technical ceramics. *Journal of Materials Science* **47**, 4211–4235 (2012).

50. Fu, J., Song, T., Liang, X. & Zhao, G. First-principle studies of phonons and thermal properties of aln in wurtzite structure. *Journal of Physics: Conference Series* **574**, 012046 (2015).

51. Pan, T. *et al.* Enhanced thermal conductivity of polycrystalline aluminum nitride thin films by optimizing the interface structure. *Journal of Applied Physics* **112** (2012).

52. Dalla Longa, F., Kohlhepp, J., De Jonge, W. & Koopmans, B. Influence of photon angular momentum on ultrafast demagnetization in nickel. *Physical Review B* **75**, 224431 (2007).

53. Rouzegar, R. *et al.* Laser-induced terahertz spin transport in magnetic nanostructures arises from the same force as ultrafast demagnetization. *Phys. Rev. B* **106**, 144427 (2022). URL `https://link.aps.org/doi/10.1103/PhysRevB.106.144427`.

54. Zhang, W. *et al.* Ultrafast terahertz magnetometry. *Nature Communications* **11** (2020).

55. Beens, M., Duine, R. A. & Koopmans, B. $s-d$ model for local and nonlocal spin dynamics in laser-excited magnetic heterostructures. *Phys. Rev. B* **102**, 054442 (2020). URL `https://link.aps.org/doi/10.1103/PhysRevB.102.054442`.
